# Estimating pregnancy rate from blubber progesterone levels of a blindly biopsied beluga population poses methodological, analytical and statistical challenges

**DOI:** 10.1093/conphys/coad075

**Published:** 2023-09-26

**Authors:** L -A Renaud, X Bordeleau, N M Kellar, G Pigeon, R Michaud, Y Morin, S Lair, A Therien, V Lesage

**Affiliations:** Department of Fisheries and Oceans Canada, Maurice Lamontagne Institute, P.O. Box 1000, 850 Route de la Mer, Mont-Joli, Québec, G5H 3Z4, Canada; Department of Fisheries and Oceans Canada, Maurice Lamontagne Institute, P.O. Box 1000, 850 Route de la Mer, Mont-Joli, Québec, G5H 3Z4, Canada; Southwest Fisheries Science Center, National Marine Fisheries Service, P.O. Box 271, La Jolla, California 92038, USA; Institut de recherche sur les forêts, Université du Québec en Abitibi-Témiscamingue, Rouyn-Noranda, Québec, J9X 5E4, Canada; Groupe de recherche et d’éducation sur les mammifères marins (GREMM), 108 de la Cale-Sèche, Tadoussac, Québec, G0T 2A0, Canada; Department of Fisheries and Oceans Canada, Maurice Lamontagne Institute, P.O. Box 1000, 850 Route de la Mer, Mont-Joli, Québec, G5H 3Z4, Canada; Faculté de médecine vétérinaire, Université de Montréal, P.O. Box 5000, 3200 Rue Sicotte, St-Hyacinthe, Québec, J2S 7C6, Canada; Department of Fisheries and Oceans Canada, Maurice Lamontagne Institute, P.O. Box 1000, 850 Route de la Mer, Mont-Joli, Québec, G5H 3Z4, Canada; Department of Fisheries and Oceans Canada, Maurice Lamontagne Institute, P.O. Box 1000, 850 Route de la Mer, Mont-Joli, Québec, G5H 3Z4, Canada

**Keywords:** Beluga, biopsies, blubber progesterone, mixture model, pregnancy

## Abstract

Beluga (*Delphinapterus leucas*) from the St. Lawrence Estuary, Canada, have been declining since the early 2000s, suggesting recruitment issues as a result of low fecundity, abnormal abortion rates or poor calf or juvenile survival. Pregnancy is difficult to observe in cetaceans, making the ground truthing of pregnancy estimates in wild individuals challenging. Blubber progesterone concentrations were contrasted among 62 SLE beluga with a known reproductive state (i.e. pregnant, resting, parturient and lactating females), that were found dead in 1997 to 2019. The suitability of a threshold obtained from decaying carcasses to assess reproductive state and pregnancy rate of freshly-dead or free-ranging and blindly-sampled beluga was examined using three statistical approaches and two data sets (135 freshly harvested carcasses in Nunavik, and 65 biopsy-sampled SLE beluga). Progesterone concentrations in decaying carcasses were considerably higher in known-pregnant (mean ± sd: 365 ± 244 ng g^−1^ of tissue) than resting (3.1 ± 4.5 ng g^−1^ of tissue) or lactating (38.4 ± 100 ng g^−1^ of tissue) females. An approach based on statistical mixtures of distributions and a logistic regression were compared to the commonly-used, fixed threshold approach (here, 100 ng g^−1^) for discriminating pregnant from non-pregnant females. The error rate for classifying individuals of known reproductive status was the lowest for the fixed threshold and logistic regression approaches, but the mixture approach required limited *a priori* knowledge for clustering individuals of unknown pregnancy status. Mismatches in assignations occurred at lipid content < 10% of sample weight. Our results emphasize the importance of reporting lipid contents and progesterone concentrations in both units (ng g^−1^ of tissue and ng g^−1^ of lipid) when sample mass is low. By highlighting ways to circumvent potential biases in field sampling associated with capturability of different segments of a population, this study also enhances the usefulness of the technique for estimating pregnancy rate of free-ranging population.

## Teaser

We validated the use of blubber progesterone to estimate the probability of pregnancy in beluga females. Samples from individuals of known reproductive status, and from hunted or free-ranging individuals of unknown reproductive status, were used to compare the influence of lipid content, sample mass, and the statistical approach on a female’s probability of pregnancy.

## Introduction

Pregnancy rate is a measure of fecundity that can provide valuable information about perinatal mortality, abortion and fetal resorption depending on when sampling is done. At the individual level, pregnancy is commonly determined by visual (Miller *et al.,* 2012), ultrasound (Steinman *et al.,* 2012) or reproductive tracts examination (Mansour *et al.,* 2002), or by progesterone quantification (Gardiner *et al.,* 1996). This hormone is a lipophilic sex steroid produced by the *corpus luteum* and the primary progestogen source for the onset and maintenance of pregnancy (Pineda, 2003). Tissues for progesterone quantification commonly include urine and blood (Robeck *et al.,* 1993, 2005). In wild species for which capture is not possible or poses logistical or ethical challenges such as cetaceans, there has been an increased interest in quantifying progesterone from tissues or samples that can be collected remotely such as sub-epidermal blubber (Kellar *et al.,* 2006; Trego *et al.,* 2013), blow (Hogg *et al.,* 2009; Richard *et al.,* 2017) and feces (Rolland *et al.,* 2005; Hunt *et al.,* 2019). In all cases and sample matrices, measured progesterone levels need to be validated against a known reproductive status for the method to be useful at assessing pregnancy status of individuals and pregnancy rate of a population.

Several methods have been proposed for such validation using species amenable to manipulation or direct observation of reproductive status, including comparison across tissues from individuals of known status (Goertz *et al.,* 2019) and ultrasound imaging (Wells, 2014; Kellar *et al.,* 2017). In free-ranging species, direct observation of pregnancy or of an offspring with a recognizable individual some time following sampling is necessary for such validation (Kellar *et al.,* 2017; Kershaw *et al.,* 2021). In carcasses from dead animals, the presence of a *corpus luteum* in the ovaries, or of a fetus or fetuses in the reproduction tract can provide the grounds against which to compare progesterone levels in various tissues, given the stability of this hormone over time, even in decaying tissues (Kellar *et al.,* 2006). Blubber progesterone levels typical of pregnancy have been established for a number of cetacean species using carcasses, biopsy sampling (e.g. 35 ng g^−1^; Goertz *et al.,* 2019) or direct observation of calves the following year (Pallin *et al.,* 2018; Kershaw *et al.,* 2021). While some of these studies have relied on beach-cast carcasses, direct harvests or fishery bycatch to validate reproductive status (Mansour *et al.,* 2002; Kellar Nicholas *et al.,* 2013; Trego *et al.,* 2013), most established pregnancies by pairing hormone biomarkers with sighting history (Pallin *et al.,* 2018; Kershaw *et al.,* 2021). However, whether blubber progesterone levels determined from carcasses can be used to interpret levels in blubber biopsies is unknown.

Although an individual reproductive state may be of interest in longitudinal studies of individual fitness (e.g. North Atlantic right whale *Eubalaena glaciali*s; (Rolland *et al.,* 2005)), it is often only a proximate means for assessing pregnancy rate of a population, an important parameter for conservation. For such an estimation to be accurate, sampling design for biopsy field collections must ensure equal availability to sampling for all age and sex classes and reproductive statuses. This may be particularly challenging in species where males and females are not size-dimorphic, or for which age or sex segregation or differential behaviour is expected (e.g. Kellar *et al.,* 2013). Finally, gestation in several cetacean species exceed one year, leading to a temporal overlap between new- and late-pregnancies (Goertz *et al.,* 2019). For these species, sampling design must carefully consider sampling timing. In this study, we explore how these many challenges can be addressed using a small cetacean as a case study.

Beluga in the St. Lawrence Estuary (SLE), Canada, are listed as endangered under the Species at Risk Act. Their population, which was stable or slightly increasing before 2000, initiated a decline at an annual rate of approximately 1% to an estimated 889 individuals when last assessed in 2012 (Mosnier *et al.,* 2015). Beluga carcasses from a long-term monitoring program in the Estuary and Gulf of St. Lawrence indicates a deterioration of body condition in both males and females since the late 1990s (Bernier-Graveline *et al.,* 2021), a substantial increase in the number of newborn calves reported dead since 2008 (Lesage, 2021) and a concomitant anomalous rate of peripartum complications among dead adult females (Lair *et al.,* 2016). The exact cause for this apparent reproductive failure is unclear and may be directly or indirectly linked to climate warming and local stressors such as high toxic substance burdens, low prey quality or availability and chronic exposure to noise and disturbance from shipping, whale watching and recreational activities (DFO, 2012, 2014; Lesage, 2021; Williams *et al.,* 2021).

The SLE population, similar to other beluga, exhibits age and sex segregation during summer (Michaud, 1993). They are size-dimorphic, with males being slightly larger and bulkier than females (Vladykov, 1944; Luque and Ferguson, 2010). Generally, adult males occupy deeper, colder and more offshore waters than females with calves, which tend to use shallower, warmer and more brackish waters (Michaud, 1993; Smith *et al.,* 1994; Loseto *et al.,* 2006). Calves are born with a brown colour. They turn dark grey after a year or two and progressively lighten in colour as they approach sexual maturity. Timing of turning white, however, varies with sex and among individuals and is not a good indicator of sexual maturity (Sergeant, 1973; Doidge, 1990), which is reached at age 4 to 7 years in adult females, and age 7 to 8 years in adult males (Brodie, 1971; Sergeant, 1973; Doidge, 1990; Heide-Jørgensen and Teilmann, 1994). Timing of calving varies among and within populations, but generally occurs between April and October (Sergeant, 1973; McGuire *et al.,* 2020; Shelden *et al.,* 2020) after an ~ 14 mo gestation. Lactation may last up to 2 years (Sergeant, 1973; Burns and Seaman, 1986; Matthews and Ferguson, 2015) and may overlap the following gestation (Sergeant, 1973; Doidge, 1990; McGuire *et al.,* 2020). Mating likely occurs in the spring in the St. Lawrence Estuary, with a majority of newborn calves being born in July (Vladykov, 1944; Sergeant, 1986, Béland *et al.,* 1990; Michaud, 2007). With a reproduction cycle estimated at three years in this species, approximately one-third of the sexually mature females are expected to enter gestation each year (Mosnier *et al.,* 2015). This reproductive cycle has been established for generally healthy beluga populations (Vladykov, 1944; Doidge, 1990; Heide-Jørgensen and Teilmann, 1994; Suydam, 2009). Confirmation that such a ratio is reached for SLE beluga would eliminate fetal resorption and low mating success as potential causes for low recruitment, leaving late abortion, unsuccessful weaning and low juvenile survival as alternate potential causes.

Using SLE beluga and our study objective of estimating pregnancy rate in this endangered and declining population, this study aims to highlight the challenges associated with assigning reproductive status of blindly-sampled cetaceans, comparing progesterone levels obtained from carcasses and biopsies and designing a field study that ensures accuracy in pregnancy rate estimation. Our specific objectives are to: 1) validate the blubber progesterone concentration associated with pregnancy in beluga using carcasses from females of known reproductive status (pregnant, resting, lactating); 2) assess the performance of three statistical approaches to threshold determination or pregnancy assignment; 3) determine pregnancy rate for the SLE population and a sample of the Nunavik Inuit subsistence harvest using blindly-sampled individuals via biopsy and fresh carcasses, respectively; 4) examine the variability in lipid contents in blubber samples collected from carcasses and through biopsies; 5) assess the impacts of reporting unit for progesterone concentration (per tissue weight vs. lipid weight) on estimated pregnancy rates; and 6) provide a statistical approach to address uncertainty in sexual maturity and pregnancy rates when sampling wild populations of unknown reproductive status.

## Material and Methods

This study used three sample groups: SLE_carcasses_, NUN_carcasses_ and SLE_biopsies_ (n = 62, 135 and 65, respectively), collected via the SLE carcass recovery program, Nunavik subsistence harvests and biopsy sampling of the free-ranging SLE population. SLE_carcasses_ were from individuals with known pregnancy status, whereas NUN_carcasses_ and SLE_biopsies_ were from individuals of unknown pregnancy status. We built a series of models using the SLE_carcasses_ samples with known pregnancy status and then implemented the models on the NUN_carcasses_ and SLE_biopsies_ samples to estimate pregnancy rates from these two samples with no reproductive state information attached.

### Data sources for pregnancy assessment

#### Carcass-based programs

##### St. Lawrence estuary necropsies

Well- to moderately-well preserved SLE beluga carcasses (code 2—3; Geraci and Lounsbury, 1993) necropsied from 1997—2019 at the Veterinary Medecine Faculty from University of Montreal were used to estimate the blubber progesterone (BP) threshold associated with pregnancy. Sex and sexual maturity were confirmed primarily from reproductive tract morphology and degree of development. For females, reproductive status was also assessed from asymmetrical distension of uterus, presence of a fetus, ovarian *corpora lutea* and presence of milk in the mammary glands, indicative of an active lactation. Markedly distended asymmetric uterus with open cervix was interpreted as recent (few days) parturition. Females with mildly to moderately asymmetric uterus without cervical mucus plug were considered to have giving birth two to four week prior to their death. Small symmetrical uterus without the presence of a fetus was seen in non-pregnant females or females that have not giving birth in the two months prior to the stranding. On total (n = 62), our SLE_carcasses_ sample included 22 non-pregnant individuals (i.e. 11 males, four sexually immature females and seven mature but non-pregnant females), 13 pregnant females, 17 lactating females (evidence of active lactation and no signs of recent birth) and ten females that had recently given birth and in very early stage of lactation. Among the 13 pregnant females, 10 were full-term pregnant females (died from dystocia or postpartum complications; Lair *et al.,* 2016), one was newly pregnant (small fetus present, in the first few weeks of pregnancy) and two had no specific mention on the stage of pregnancy and were no longer available for retrospective examination. Age was estimated from counts of dentinal growth layer groups following Lesage *et al.* (2014) and used only to identify potential misclassification among sexual maturity stages. A 10 cm × 10 cm sample comprised of skin, blubber and underlying muscle was obtained from each carcass, sealed in aluminum foil rinsed with hexane, archived at −20°C and later subsampled in its core (to avoid oxidation) to obtain ~ 100 mg wet weight of the blubber outer layer, i.e. the layer available when biopsy sampling.

#### Nunavik subsistence harvests

To examine potential effects of carcass freshness on BP concentrations, BP concentrations were also quantified in 135 freshly-killed females accessed through the Nunavik Inuit subsistence hunts in Hudson Bay and Hudson Strait, Canada, in 2001—2019. All NUN_carcasses_ samples were from females of unknown reproductive status, with sex confirmed genetically (as per Bérubé and Palsbøll, 1996; Shaw *et al.,* 2003). Most (122/135) females were assumed to be sexually mature (8–49 years-old; (Stewart *et al.,* 2006; Ferguson *et al.,* 2020)). Similar to SLE carcasses, an archived skin/blubber/muscle sample preserved at −20°C was subsampled to obtain 100 mg wet weight of blubber from the outer layer for steroid extractions.

#### Biopsy-based program: St. Lawrence estuary

Determining whether pregnancy rate of free-ranging SLE beluga deviated from the expected one-third annual ratio required that a random and unbiased sample of sexually mature females be obtained from this population. In this perspective, there was a need for all females to be equally available to sampling, regardless of their habitat use, reproductive status or sexual maturity. Our incapacity to confirm readily sex and maturity from size and colour made this field sampling design challenging.

**Figure 1:**
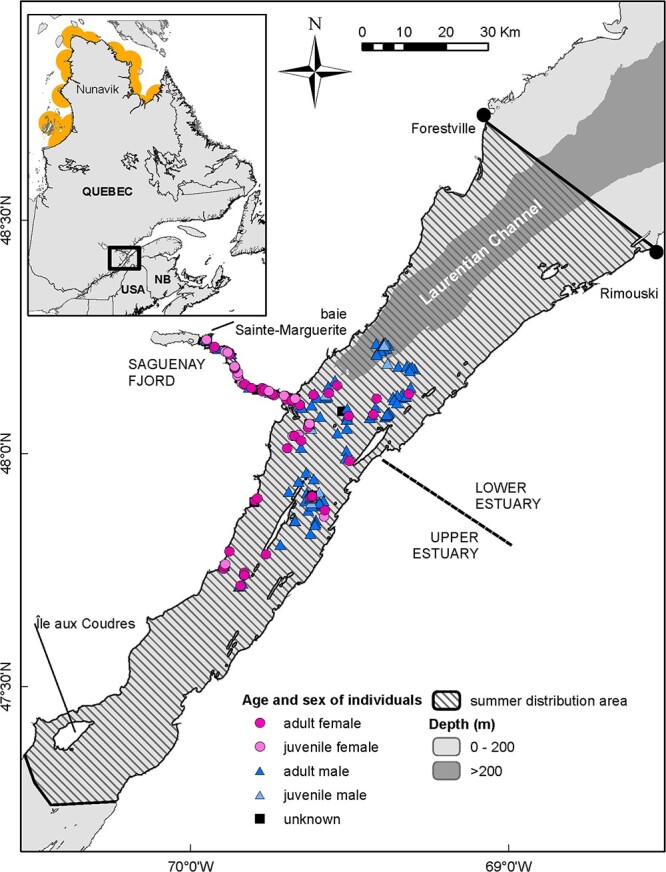
Portions of the St. Lawrence Estuary and Saguenay River (Quebec, Canada) included in this study and showing spatial age- and sex-segregation in beluga. Locations of Nunavik Inuit subsistence hunts in Hudson Bay and Hudson Strait, Canada, are in inset. Depth data are from Canadian Hydrographic Service.

The SLE beluga summer distribution extends from Ile-aux-Coudres to Rimouski/Forestville, including the Saguenay Fjord up to Saint-Fulgence (Fig. 1; Mosnier *et al.,* 2010), and is characterized by a well-documented sex- and age-segregation (Michaud, 1993; Ouellet *et al.,* 2021). Generally, herds of moderately large white animals (presumed to be adult females), calves and small juveniles of both sexes use the warmer and shallower waters of the Upper SLE and the south shore portion of the Lower SLE (Fig. 1). Large white animals (presumed to be adult males) are found primarily in the Lower SLE (in both deep and shallower waters), whereas older juveniles (grey to whitish individuals) occupy all sectors. All types of herds use the Saguenay Fjord. Our sampling area did not reach the two extremes of the summer range (Fig. 1); however, a habitat connectivity analysis indicates that beluga move between these extremes and the core of the summer range where our sampling took place (Ouellet *et al.,* 2021), suggesting that no specific segment of the female population was likely missed in our sampling.

Biopsy sampling was attempted on the first individual surfacing in an adequate position to be darted. Only calves and dark grey animals of a size compatible with age 3 years or less were excluded from the sampling scheme. This ensured that all potentially mature females were included regardless of colour. However, an undetermined number of immature individuals were sampled. Calves tend to unpredictably change their flanking position around a female (Hill *et al.,* 2017), making biopsy collection riskier on lactating females. To avoid a potential sampling bias, females that were flanked repeatedly by a newborn calf, that presented themselves first in an adequate position for sampling and that would have been darted if ignoring the calf, were considered as ‘lactating females’. These ‘virtually-biopsied’ females were accounted for when estimating pregnancy rates and were pooled with other females flanked by a newborn calf and successfully biopsied. Newborn calves were easily distinguishable from one-year-old calves in the field by their erratic surfacing patterns when breathing, their tendency to project themselves out of the water, as well as from their fetal folds (Michaud, 2014). Sampling covered 4 years (2013—2016) and was conducted each year over a 3-week period in September. Timing ensured that pregnancies initiated in the spring (mating season) were well underway with expected high BP levels (Steinman *et al.,* 2012), and that females that had given birth during summer (between late June and early August; R. Michaud, unpublished report^1^) were given 1—2.5 months for their BP concentrations to decline.

Biopsies from free-ranging SLE beluga (hereafter, the SLE_biopsies_ data set) were acquired remotely (15—20 m according to range finder measurements) by firing a hollow stainless-steel dart (35 mm long and 8 mm in diameter) from a 9 m boat using a CO_2_-powered rifle with adjustable pressure (J.M. Special 25 model, DAN-INJECT). All samples were taken from an area within ~ 50 cm of the dorsal ridge. Colour and size (e.g. small grey, larger grey, almost-white, white) of individuals were used to estimate age class in the field, but no other demographic information (e.g. animal identity, age, maturity, calving history) was available at the time of sampling. While some aging methods using fatty acids and DNA methylation exist for free-ranging beluga, they remain too imprecise to help assess the probability of sexual maturity in large grey, almost-white and small white animals (Marcoux *et al.,* 2015; Bors *et al.,* 2021). The blubber layer was separated from the epidermis/dermis directly in the field using a scalpel, sealed in hexane-rinsed aluminum foil and a Cryovial™ tube, then flash-frozen in liquid nitrogen and stored at -80°C. Sex was determined molecularly using a standard PCR assay (Palsbøll *et al.,* 1992). Only females (N = 65, plus 9 virtual biopsies) were analysed for their BP concentrations.

### Sample processing

Hormone extractions were carried out following Kellar *et al.* (2006) and Goertz *et al.* (2019) with some modifications (see Appendix A of the supplementary material for detailed protocol steps). Briefly, blubber samples were mechanically homogenized and tissue debris was removed in a series of ethanol (100%), ethanol:acetone (4:1) and diethyl ether (100%) washes. After each solvent rinse, the supernatant was recovered and evaporated, and the resulting lipid residue was resuspended twice in acetonitrile and hexane, each time forming two immiscible phases: a bottom phase containing hormones in acetonitrile and an upper hexane phase containing lipids. The acetonitrile layers of both series were combined, dried down and stored at −20°C until progesterone analysis. The upper hexane layers of both series were combined and transferred to a pre-weighed dish and evaporated under a flow-hood. Following hexane evaporation, the lipid residue was weighed to determine the initial lipid content of the blubber sample.

BP was quantified using the competitive enzyme-linked immunosorbent assay (ELISA) kit from Enzo Life Sciences (ADI-900-011). The morning of the immunoassay, duplicate samples were prepared by re-suspending the progesterone extract in 500 μL of assay buffer (ADI-80-0010, Enzo Life Sciences, Farmingdale, NY, USA). The assay detection limits were between 15.6 and 500 pg/mL. Samples with concentrations outside these limits were diluted by factors of 5 to 5000 (see Appendix A of the supplementary material for details). Samples from the three data sets were not assayed in a single batch. Inter-assay variation was determined by including an internal control on each plate treated: a male sample as a negative control, and a gestating female as a positive control. Duplicate samples were used to calculate intra-assay variation. The intra-assay and inter-assay coefficients of variation were < 15% for all data sets. Extraction efficiency was measured by spiking three diluted assay standard (25, 100 and 500 ng mL^−1^ progesterone) to assay buffer, and as the mean for the three controls. Mean extraction efficiency was 88.3% for SLE_carcasses_ and NUN_carcasses_ data sets and 71.0% for the SLE_biopsies_ data set. Final progesterone concentrations were adjusted by dividing values by this mean progesterone recovery factor, as well as for dilution factor, initial blubber mass (expressed in ng g^−1^ of blubber) and sample lipid content (expressed in ng g^−1^ of lipids).

### Statistical analyses

#### Probability of pregnancy

BP concentrations were log-transformed prior to all analyses for consistency and easier comparisons between results. BP concentrations were first examined for differences among known reproductive status using an ANOVA and post-hoc Tukey tests. Our initial hypothesis was that pregnant females would have significantly larger BP concentrations than individuals in any other reproductive state. The probability of pregnancy as a function of log-transformed BP concentration was then assessed using three data sets (SLE_carcasses_ = 62, NUN_carcasses_ = 135, SLE_biopsies_ = 65) and three different approaches: i) a fixed threshold, ii) a mixture of Gaussian distributions (hereafter, mixture models) and iii) a Bayesian logistic regression (hereafter, logistic). The fixed threshold approach used a break in the BP concentration frequency distribution for discriminating pregnant from non-pregnant females. Exploratory analyses revealed the distribution of natural log-transformed BP concentrations to be multimodal, likely reflecting clusters of individuals in different reproductive states. Consequently, a second approach was applied to each data set separately, where mixtures of two or three normal distributions were fitted to log-transformed BP values. Mixture models provide a model-based approach to clustering and has the advantage of having few *a priori* assumptions on data distribution (Hamel *et al.,* 2017). Mixture models were employed to model the probability of being pregnant based on progesterone concentrations, using log-transformed BP values. These models estimate the likelihood of an individual belonging to a specific cluster using a latent grouping variable, along with the means and standard deviations of normal probability density functions for each cluster. Mixture models have been successfully applied in similar studies of blue (*Balaenoptera musculus*) and gray (*Eschrichtius robustus*) whales (Melica *et al.,* 2021a, 2021b).

Our analyses were conducted within a Bayesian framework, using Monte Carlo Markov Chains (MCMC) algorithms to estimate the parameters of the models. Analyses were conducted using the NIMBLE library (v. 0.12.0 (de Valpine *et al.,* 2017, 2022)) and R software (v. 4.0.4, (R Core Team, 2021)). The determination of the number of clusters in each mixture model was guided by the widely applicable information criterion (WAIC) since more robust approaches such as cross-validation were impossible with our sample size (Vehtari *et al.,* 2017). Weakly informative prior distributions were employed for all model parameters. Each analysis was ran using 50 000 iterations of three independent chains, with a thinning of 40 and a burn-in of 10 000 iterations. This resulted in 1000 iterations per chain to define posterior distributions of model parameters. Convergence and mixing of the chains for the means and standard deviations of each distribution was visually assessed using trace plots of the samples and Gelman-Rubin diagnostics (Gelman and Rubin, 1992). Once the means and standard deviations of each cluster were determined, each individual with an unknown reproductive status (NUN_carcasses_ and SLE_biopsies_) was assigned a posterior probability of belonging to each distribution. We assumed that the distribution with the highest mean BP levels corresponded to pregnant females. Female reproductive status (i.e. presumed pregnant or presumed non-pregnant) was assigned based on the most probable cluster.

The relationship between progesterone levels and pregnancy status was assessed by fitting a generalized linear regression with a logit link function in a Bayesian framework. The analysis was conducted in R using SLE_carcasses_ samples, where pregnancy state served as the Bernouilli-distributed response variable (pregnant versus non-pregnant), and log-transformed BP concentrations were used as predictor variables (Kellar *et al.,* 2017). Missing values in Bayesian analyses can be estimated directly, allowing us to predict the probability of pregnancy for individuals of unknown reproductive status. Males, lactating, immature or resting females were included as non-pregnant, and all confirmed pregnant females were coded as pregnant. The 10 SLE_carcasses_ females found dead shortly after giving birth (prior to entering lactation or soon after its onset) were considered as unknown reproductive status for which the reproductive status was to be predicted since their levels of BP would be expected to be anywhere in between those of pregnant and non-pregnant females.

A logistic regression was first performed on the NUN_carcasses_ data set, then on the SLE_biopsies_ data set (each model being informed by the SLE_carcasses_). However, an analysis pooling samples from all three data sets gave comparable results and are available in Appendix A. Model output parameters and 95% Bayesian credibility envelope were used to obtain a marginal posterior probability distribution of being pregnant for each female of unknown reproductive status. A mean probability estimate < 50% was assumed to indicate non-pregnancy whereas a value ≥50% was considered an indication of pregnancy. Similar to the mixture models, MCMC algorithms were used to estimate the parameters of the models within the NIMBLE library [v. 0.12.0 (de Valpine *et al.,* 2017, 2022)] and R software [v. 4.0.4, (R Core Team, 2021)]. Models were based on 50 000 iterations of three independent chains, with a thinning of 40 and a burn-in of 10 000 iterations. Convergence and mixing of the chains were assessed visually using trace plots of the samples and Gelman-Rubin diagnostics.

#### Metrics and effect on probability of pregnancy

BP concentrations were reported in ng per g of blubber tissue and in ng per g of lipid to reflect potential differences in lipid composition of samples obtained through biopsy and carcass sampling. Lipid percentages were available for all SLE_biopsies_ and NUN_carcasses_, but only for 18 of the 62 SLE_carcasses_ samples. The mean lipid percentage from these 18 samples (66.4%) was applied to SLE_carcasses_ samples with unknown lipid contents to obtain a concentration per lipid mass. Both metrics were used to estimate the probability of pregnancy using the three approaches described above.

**Table 1:** Blubber progesterone concentrations for beluga of known and unknown reproductive status, coming from the St. Lawrence Estuary (SLE_carcasses_), the harvest in Hudson Bay and Hudson Strait (NUN_carcasses_) and free-ranging beluga from the St. Lawrence Estuary (SLE_biopsies_)

**Reproductive status**			**Sample size**	**Blubber progesterone (ng g** ^**−1**^ **of tissue)**			**Significant differences between groups**	
				Average	Range	SD		
**SLE**_**carcasses**_								
**Males**		Immature	6	1.1	0.1–3.5	1.3	a^a^	
		Mature	5	0.8	0.1–1.8	0.7	a	
**Females**	Pre-gestation	Immature	4	0.6	0.3–1.3	0.5	a	
		Mature, resting	7	3.1	0.2–11.9	4.5	b	d^b^
	Gestation	Pregnant	13	365	9.0–878	244		c
	Post-parturition	Recent parturition	10	273	4.8–695	227		c
		Lactating	17	38.4	0.2–413	100		d
**NUN**_**carcasses**_								
		Unknown	135	231	0.1–1203	297		
**SLE**_**biopsies**_								
		Unknown	65	98	0.3–464	127		

For the SLE_carcasses_, significant differences are based on post-hoc Tukey tests for multiple comparisons. SD: standard deviation.

a Based on a nested two-way ANOVA of log-transformed progesterone concentration using sex (male, female) and sexual maturity (mature, immature) as independent variables. Significant differences among groups (different letters) are based on a post-hoc Tukey test for multiple comparisons.

b Based on a one-way ANOVA of log-transformed progesterone concentration using reproductive status (pregnant, resting, recently parturient, lactating) as independent variables. Significant differences among groups (different letters) are based on a post-hoc Tukey test for multiple comparisons.

#### Estimating pregnancy rate from blindly-sampled populations

To estimate pregnancy rate, one needs to determine the proportion of mature females in the sample. The potential bias introduced by the mismatch between timing of turning white and sexual maturity in beluga was assessed by comparing pregnancy rates in the SLE_biopsies_ data set to ‘theoretical’ pregnancy rates per colour class in the SLE_carcasses,_ selecting 92 females of known colour class and reproductive status or sexual maturity (mature, immature), and aged > 3 years (to match field sample). This comparative analysis was performed using the mixture model approach with three Gaussian distributions (see Results).

## Results

### Probability of pregnancy

BP concentrations in SLE_carcasses_ varied between sex and maturity classes (nested two-way ANOVA, F_2,58_ = 5.0; *P* < 0.05) and were higher in mature females compared to males (mature or immature) or immature females (post-hoc Tukey’s tests; all *P* < 0.05; Table 1). BP concentrations of females, in ng g^−1^ tissue, also varied according to reproductive state (one-way ANOVA, F_3,43_ = 255, *P* < 0.05), and were the highest in pregnant and recently parturient females with averages of 365 and 273 ng g^−1^ of tissue, respectively (post-hoc Tukey’s test, all *P* < 0.05; Table 1). These levels were significantly higher than those observed in resting or lactating females (both *P* < 0.05). Lactating females presented intermediate BP values, that were not statistically different from those from mature but resting females (*P* = 0.5).

**Figure 2:**
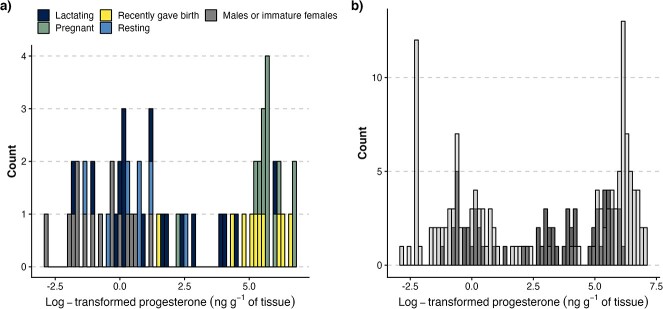
Blubber progesterone concentration (ng per gram of tissue) in beluga samples from the a) SLE_carcasses_ and b) combined NUN_carcasses_ and SLE_biopsies_ programs. In panel a), colours indicate reproductive status. In panel b), NUN_carcasses_ and SLE_biopsies_ are shown as light and dark grey bars, respectively. Raw progesterone concentrations were log-transformed.

The range of BP concentrations was comparable for SLE_carcasses_ and NUN_carcasses_, with all but 4 samples in the latter being less than 873 ng g^−1^ tissue (Table 1; Fig. 2, Appendix A, Fig. A.1). However, SLE_biopsies_ presented approximately half the range of BP values compared to carcasses, likely due to lower lipid contents (see below, Fig. A.2). An analysis of variance of lipid percentage (continuous variable) as a function of sampling program (a three-level factor) revealed significant differences in lipid content between the three programs (F_2,214_ = 179, *P* < 0.001).

A visual inspection of the distribution of BP values among the three data sets suggests a natural break in the data at around 100 ng g^−1^ of tissue, with no BP values between 85.9 and 114 ng g^−1^ tissue in any of the data sets. The use of a 100-ng g^−1^ tissue (fixed) threshold led to the misclassification in SLE_carcasses_ of one newly pregnant female (BP: 9.0 ng g^−1^ tissue, classified as non-pregnant) and one lactating female (BP: 413 ng g^−1^ tissue, presumed pregnant). Eight females that had recently given birth (BP range: 120–695 ng g^−1^ tissue) were classified as pregnant using this approach, while two (BP range: 4.8–73 ng g^−1^ tissue) were classified as non-pregnant (Table B.1). The time lag necessary for blubber progesterone to decline after parturition being unknown but likely a few weeks, we assumed that females that recently gave birth were correctly classified as pregnant based on their progesterone profiles at the time of sampling. The fixed threshold thus led to a total error rate of four misclassifications over 62 individuals.

**Figure 3:**
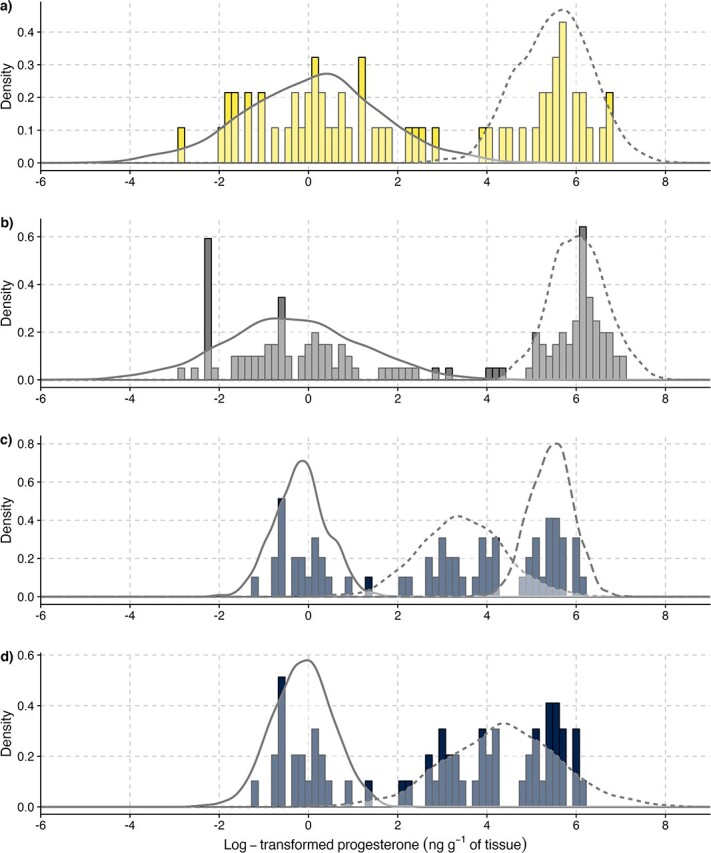
Mixtures of Gaussian distributions used to assign a probability of being pregnant in beluga based on log-transformed blubber progesterone concentration. Panel a) shows distributions for necropsied beluga from the St. Lawrence Estuary, Canada (SLE_carcasses)_; panel b) shows beluga harvested in Hudson Bay and Hudson Strait, Canada (NUN_carcasses)_; panels c) and d) show free-ranging beluga biopsied in the St. Lawrence Estuary, Canada (SLE_biopsies_) with samples respectively clustered into three and two groups. Means, standard deviations of each distribution and their respective 95% lower and upper credibility intervals, are given for each data set in Table A.1. The description of the different reproductive statuses observed in SLE_carcasses_ is shown on Figure 2. Histograms were scaled between 0 and 1 to match the scale of the density plots.

Results from the mixture models confirmed that log-transformed BP concentrations for the SLE_carcasses_ and NUN_carcasses_ data sets were better represented by two clusters, namely a ‘low’ and a ‘high’ progesterone cluster, whereas for SLE_biopsies_, they were best represented by three clusters (i.e. a ‘low’, an ‘intermediate and a ‘high’ progesterone cluster; WAIC 194 *vs* 145; Fig. 3, Tables A.1, A.2). We could also force the use of a two-cluster model for the SLE_biopsies_ similar to the SLE_carcasses_, but this resulted in the merging of the ‘intermediate’ and ‘high’ progesterone clusters, decreasing the overall threshold to distinguish the ‘low’ and ‘high’ clusters below biological reasonable progesterone levels for pregnancy. Using this approach, SLE_carcasses_ samples of 55 ng g^−1^ tissue (4.0 ng g^−1^ tissue, log-transformed) had a ~ 80% probability of being classified as pregnant, while samples of 17 ng g^−1^ had a ~ 10% probability of being pregnant. While we assumed that the low and high progesterone clusters respectively corresponded to non-pregnant and pregnant females, the model classified a newly pregnant female in the low-progesterone cluster (BP value: 9.0 ng g^−1^ tissue) and classified four lactating females (BP range: 47–413 ng g^−1^ tissue) and nine females that recently gave birth (BP range: 73–695 ng g^−1^ tissue) in the high progesterone cluster (Tables 2, A.3, B.1). Assuming that the pregnancy signal of females that recently gave birth reflected pregnancy a few weeks prior to sampling, the mixture approach led to a total error rate of six misclassifications. The mixture model results still confirmed that this approach was robust, with pregnant females from SLE_carcasses_ correctly assigned to their known reproductive status with posterior probabilities of over 90%. Rerunning the model without the males provided similar results (not shown). However, small sample size precluded us from assessing the fit of the model using a more robust train-validate-test approach.

**Table 2:** Classification of individuals of known (SLE_carcasses_) and unknown (NUN_carcasses_ and SLE_biopsies_) reproductive status based on progesterone concentrations (in ng g^−1^ of tissue), made using three statistical approaches: a fixed threshold, a model-based clustering and a logistic regression

	100 ng g^−1^ Fixed threshold		Mixture model		Logistic	
	N	Mean ± SD	N	Mean ± SD	N	Mean ± SD
**SLE**_**carcasses**_ ^**a**^						
Presumed non-pregnant (49)	41	8.8 ± 19.8	37	2.7 ± 3.8	39	5.2 ± 11.5
Presumed pregnant (13)	21	371 ± 215	25	323 ± 227	23	346 ± 222
**NUN**_**carcasses**_						
Presumed non-pregnant	73	4.5 ± 13.4	71	2.6 ± 7.2	71	2.6 ± 7.2
Presumed pregnant	62	497 ± 245	64	483 ± 253	64	483 ± 253
**SLE**_**biopsies (All samples included)**_						
Presumed non-pregnant	43	18.4 ± 22.6	20	0.9 ± 0.5	38	12.3 ± 15.6
Intermediate	0	-	23	33.6 ± 21.3	0	-
Presumed pregnant	22	253 ± 99.0	22	253 ± 99.0	27	218 ± 116
**SLE**_**biopsies (Samples with < 5% lipids excluded—see Results)**_						
Presumed non-pregnant	30	19.9 ± 24.6	15	1.1 ± 0.9	27	14.6 ± 19.5
Intermediate	0	-	16	43.5 ± 28.6	0	-
Presumed pregnant	21	258 ± 98.4	20	265 ± 95.2	24	234 ± 112

For SLE_carcasses_ the known number of individuals in each category is indicated in parenthesis. The resulting number of individuals (N) in each category and associated mean progesterone concentrations (Mean ± standard deviation, SD) are given for each statistical approach. Individual probabilities appear in Supplementary Tables B 1 to B 4 of Appendix B.

a ^a^For classification of carcasses distinguishing the different stages of pregnancy and non-pregnancy, see Appendix A: Table A. 3.

When analysing pooled samples from the three data sets, a two-cluster model demonstrated the best fit to the data based on the WAIC (Fig. A.3). This model yielded consistent results for the SLE_carcasses_ and NUN_carcasses_ data sets, including the number of presumed pregnant and non-pregnant individuals and mean progesterone concentration per group (Table A.4). However, for the SLE_biopsies_, a two-cluster model led to a reclassification of most ‘intermediate’ females into the high-progesterone cluster, with 38 females assigned to the high progesterone cluster and 27 females assigned to the low progesterone cluster.

**Figure 4:**
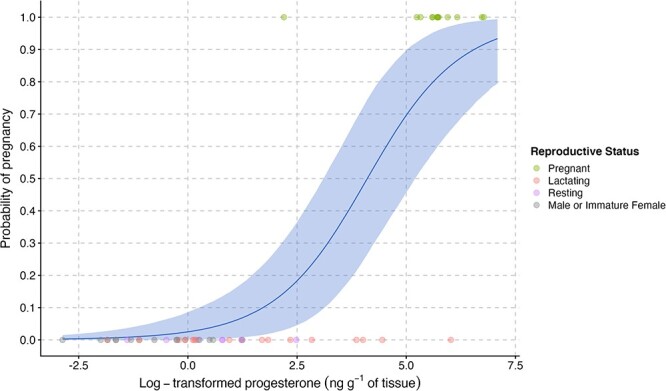
Logistic regression used to predict the probability of being pregnant in beluga based on their blubber progesterone concentration expressed in ng∙g^−1^ of tissue. The GLM used individuals of known reproductive status as input data (here, the SLE_carcasses_ data set). Individuals of unknown reproductive status were entered as ‘NA’ (here, the SLE_biopsies_) and were thus predicted by the GLM.

Predictions from the logistic approach appear in Table 2, with the odds of being pregnant increasing by 2.7 [1.8, 4.9] for every ng g^−1^ of BP and reaching equal odds at 61.0 [21.5, 189] ng g^−1^ of BP for the SLE_carcasses._ The models classified nine of the 10 recently pregnant females as pregnant (Fig. 4, Table A.3). Two lactating (BP range: 86–413 ng g^−1^ tissue) females were classified as pregnant, whereas one newly pregnant female (BP: 9.0 ng g^−1^ tissue) was classified as non-pregnant using this approach, leading to a total of four misclassifications.

The number of pregnant females varied between 62 and 64 individuals for NUN_carcasses_ among approaches (Tables 2, B.2). For the SLE_carcasses_, the number of pregnant females varied between 21 and 25 individuals (Table 2). For SLE_biopsies_, the estimated number of pregnant females was the highest for the logistic approach and equal for the fixed threshold and mixture models (Table 2). The three approaches also led to different classifications for non-pregnant females given the differences in the number of groups they allowed (two for logistic and fixed threshold approaches versus three for mixture models; Table A.2). In total, 23 females (BP range: 3.9–70.8 ng g^−1^ tissue) were included in the ‘intermediate’ cluster; 18 of them (BP range: 60.2–70.8 ng g^−1^ tissue) were classified as non-pregnant in the logistic and fixed threshold approaches, while 5 of the 23 females (BP range: 3.9–53.1 ng g^−1^ tissue) were classified as non-pregnant by the threshold approach and as pregnant using the logistic regression (Table A.2). Individual probabilities and classification according to each approach are presented in Appendix B.

#### Metrics, lipid content and effect on probability of pregnancy

Mean percent lipid was 2.6-fold lower on average in biopsies than carcasses, averaging 24.3% in SLE_biopsies_ compared to 66.4% and 60.9% in SLE_carcasses_ and NUN_carcasses_, respectively (Fig. A.2, Table B.4). In SLE_biopsies_, 41 of 65 samples had lipid contents < 30% and 19 of 65 samples < 6%, while none of the SLE_carcasses_ had lipid contents < 30% (or even 50%); only 3 of 135 of the NUN _carcasses_ data had contents below 30% lipids. Expressing BP concentrations while accounting for lipid contents increased the threshold value for discriminating pregnant from non-pregnant females from 100 to approximately 150 ng g^−1^lipid, based on examination of frequency distributions (Figs. A.1, A.2, A.4-A.6, Tables A.5, A.6). Lipid contents especially impacted the SLE_biopsies_ data, with nearly 30% of the individuals switching assignation among classes when expressing BP values per g of lipids, and this, independently of the statistical approach. All these individuals had lipids contents < 10% (Table 3, Fig. A.6). To minimize the potential effect of low lipid contents on probability of pregnancy, all SLE_biopsies_ samples with ≤ 5% (i.e. 14 out of 65) were excluded from the analyses (Tables 2, B.4). Samples with ≤ 10% lipids were not excluded because of low sample size and convergence issues.

**Table 3:** Classification of individuals of known (SLE_carcasses_) and unknown (NUN_carcasses_ and SLE_biopsies_) reproductive status using similar approaches as in Table 2, but using progesterone concentrations expressed in ng per gram of lipid and not per gram of tissue

	150 ng g^−1^ threshold		Mixture model		Logistic	
	N	Mean ± SD	N	Mean ± SD	N	Mean ± SD
**SLE**_**carcasses**_						
Presumed non-pregnant (49)	41	13.2 ± 29.6	37	4.0 ± 5.7	39	7.7 ± 17.2
Presumed pregnant (13)	21	556 ± 322	25	483 ± 340	23	518 ± 332
**NUN**_**carcasses**_						
Presumed non-pregnant	73	7.3 ± 20.8	71	4.4 ± 11.3	71	4.4 ± 11.3
Presumed pregnant	62	820 ± 448	64	798 ± 458	64	798 ± 458
**SLE**_**biopsies**_						
Presumed non-pregnant	28	32.5 ± 35.3	33	49.0 ± 72.8	26	25.6 ± 26.6
Presumed pregnant	37	782 ± 520	32	872 ± 502	39	748 ± 527

Individual probabilities appear in Supplementary Tables B 5 to B 7 of Appendix B.

#### Estimating pregnancy rate for the blindly-sampled SLE beluga population

Once the 14 individuals from SLE_biopsies_ with ≤ 5% lipids were excluded, the mixture model approach estimated pregnancy rate for the SLE beluga population at 20/60 or 30.0% when including the 9 virtually biopsied-females as non-pregnant in the ‘intermediate’ cluster. This was if considering that all biopsied individuals were mature, which is improbable. Necropsy reports for females aged 3+ years (N = 93) indicate that the proportion of mature females among the deads was 100% for white females (i.e. 75/75 of carcasses), 100% for off-white females (i.e. 4/4 carcasses) and 10% for grey individuals (i.e. 1/10 carcasses). Applying these rates of maturity to each colour class in our sample predicted ~ 49/60 (or 82%) mature females in our SLE_biopsies_ sample when adding the 9 virtually-biopsied animals as mature (lactating) females: 33 white females (30 biopsied, plus 3 virtually biopsied); 12 off-white females (100% of 9 biopsied, plus 3 virtually biopsied); and 4 grey individuals (10% of 12 biopsied, plus 3 virtually biopsied). Assuming a normal 3-year calving interval, 16 of these females would be expected to be pregnant (i.e. 33% of 49 mature females), with corresponding rates per colour class of 33% for white (1/3 of 100% mature), 33% for off-white (1/3 of 100% mature) and 3% for grey (1/3 of 10% mature) females. The minimum number of mature females in our sample was 45 (i.e. individuals deemed pregnant or in the ‘intermediate’ cluster in Table 2, plus the nine virtual and lactating females), thus lower than the value predicted (i.e. 49) based on maturity per colour class from carcasses. However, distributing the 15 non-pregnant females based on colour (seven white, three off-white and five grey individuals) and expected maturity rates resulted in an additional ~ 11 mature individuals, and a pregnancy rate shifting from 20/45 or 44.4% to 20/56 or 35.7%. Among colour classes, pregnancy rates were slightly higher than predicted for white (36.3% versus 33% predicted) and grey females (26.7% versus 3% predicted), and as predicted for off-white females (33% versus 33% predicted).

## Discussion

This study produced four key results: 1) a validation of blubber progesterone levels associated with pregnancy, highlighting differences in range of values between carcass- and biopsy-collected samples; 2) a performance analysis for three quantitative approaches to assign pregnancy probabilities, showing a similar accuracy for all approaches; 3) a comparative analysis revealing higher lipid contents in carcasses than in biopsy samples, with consequently exaggerated effects in samples with relatively low lipid content; 4) pregnancy rate estimates for a blindly-sampled, free-ranging population, including aspects of sampling design and analytical methods that can help address uncertainty in sexual maturity and reduce biases in pregnancy rate estimations. While our findings are specific to the St. Lawrence Estuary beluga population due to variations in hormone and lipid levels, the methodologies and analytical approaches we employed can serve as a useful reference for future studies facing similar challenges for estimating pregnancy from biopsy samples.

The detailed necropsy reports were invaluable to this study as they allowed individuals not only to be classified as mature or immature, but also to be assigned to specific reproductive stages and categories within these stages (e.g. lactating but recently pregnant). As a result, little ambiguity persisted in the data set, demonstrating the usefulness of BP concentrations to accurately diagnose pregnancy in beluga. Beluga exhibit reproductive seasonality, with estrous cycle occurring mainly in March to May and breeding in April to May (Robeck *et al.,* 2005; Steinman *et al.,* 2012). Elevated progesterone levels during pre-breeding and post-breeding periods and associated with pregnancy have been documented in marine and other mammals (Robeck *et al.,* 2005; Steinman *et al.,* 2012). The high BP concentrations (range: 206–878 ng g^−1^ of tissue) in all pregnant females with near full-term fetuses (1.46–1.78 m) and ten pregnant females for which dystocia was the likely cause of death (BP range: 163.4–878 ng g-1 of tissue) compared to mature and resting or immature females (range: 0.1—11.9 ng g^−1^ of tissue) suggest that high BP levels persist in the blubber during most of the gestation in beluga, similar to observations for other whale species [e.g. in killer whales (Atkinson *et al.*, 1999); bottlenose dolphins (West *et al.*, 2000)]. Therefore, there was a high likelihood that pregnant females sampled in September in our study, i.e. ~ 6 months into pregnancy, would be easily discriminated from resting or immature individuals based on BP concentrations at that time.

The three approaches to pregnancy probability estimation (e.g. fixed threshold, mixture models and logistic approach) performed similarly in the accuracy of assignments of individuals according to their (known) reproductive status. Of paramount importance, the mixture model was the sole approach that required no *a priori* knowledge for assigning pregnancy status, a clear advantage when dealing with no individuals of known reproductive status. The biological meaning of each cluster must be determined *a posteriori*, but means and standard deviations of each Gaussian distribution are available to facilitate this decision process. While raw probabilities of belonging to a cluster, especially when validated with individuals of known reproductive status, offer valuable insights in how extreme these posterior probabilities are, discrete categories are often essential from a conservation standpoint. Both raw probabilities and a *posteriori* classifications were thus presented as complementary information. In addition, the mixture model approach allows for multiple clusters of BP concentrations to be considered, not just a dichotomous classification (e.g. pregnant, not pregnant) as in the other two approaches. Indeed, natural variations in progesterone concentration are driven by the process of reproduction throughout the life cycle of beluga, making it a more complex issue than a simple binary pregnant versus non-pregnant classification of individuals solely based on progesterone profiles. For example, females that recently gave birth still showed high progesterone concentrations, resulting in a continuum of high to low progesterone concentrations and explaining the classifications of nine of them as pregnant females by the mixture models and the logistic regression. Even with successful birth, progesterone levels will decrease progressively throughout lactation (e.g. Kuhn, 1969). While mixture models are ideal for the few assumptions they make, a simple model such as used here, assigns clusters without any biological rationale. Knowledge of the species biology is still necessary to make a sound interpretation of the meaning of each cluster.

There were however discrepancies in the range of BP values between carcasses and biopsy samples and in estimated reproductive state as a result of low lipid contents, which led us to question the reliability of carcasses as ‘input’ data to assess pregnancy in individuals of unknown reproductive status. While steroid hormone concentrations are affected little by storage time (Trana *et al.,* 2015), some may show higher concentrations in more decayed carcasses as a result of an increase in quantity of extracted lipids (Mello *et al.,* 2017). The use of carcasses in preservation state ≤ 3.0 most probably limited this effect on our results (see Mello *et al.,* 2017). Blubber biopsies, in turn, might be more prone to lipid loss in the field compared to frozen carcasses (Carbajal *et al.,* 2021), resulting in lower lipid contents. The dart hit point on the whale may affect blubber extraction depending on angle and position relative to the dorsal (fibrous) crest or fin (Noren and Mocklin, 2012). However, photo examination for each biopsied beluga provided no support for such a pattern. Blubber samples were approximately 50 mg in weight while 150 mg is recommended (Kellar *et al.,* 2006, 2015), but were similar to other studies from field-biopsied whales (Goertz *et al.,* 2019; Graham *et al.,* 2021; Wittmaack *et al.,* 2022). Curiously, an earlier study of SLE beluga using similarly-sized blubber samples and an almost identical sampling design resulted in much higher lipid contents (range: 4.1 to 86.6) compared to this study (Hobbs *et al.,* 2003). In SLE beluga, a decrease in the amount of essential fatty acids present in their blubber has been documented between 1998 and 2016, which was interpreted as an apparent decline in body condition and energy stores (Bernier-Graveline *et al.,* 2021). Low lipid contents were also noted in biopsy samples obtained from another endangered beluga population (Cook Inlet) using the exact same field material as for SLE beluga (N. Kellar, *pers. comm., 13 Jan. 2022*). While there may be methodological explanations for low lipid contents in our and Cook Inlet beluga studies, a poorer body condition in recent years cannot be ignored as a potential contributing factor. Nevertheless, lower lipid contents in biopsies compared to carcasses were unlikely to affect probability of pregnancy, except at very low levels (<5%). The reporting of concentrations per gram of tissue and per gram of lipids (Carbajal *et al.,* 2021) and pregnancy assignations using both units may help identifying samples likely to provide spurious pregnancy estimates.

Pregnancy rate for the free-ranging SLE beluga was estimated at 35.7%, with rates slightly higher than expected in white females (36%), conform to expectations in off-white females (33%) and higher than expected in grey individuals (27%). The overall pregnancy rate was only slightly higher than the 32.6% [95% CI: 0.26–0.37] rate for the population estimated from inter-birth intervals for 1983–2012 (Mosnier *et al.,* 2015). While this may suggest at first glance a normal pregnancy rate for the SLE beluga population, there are a number of factors that may make pregnancy rate deviate positively or negatively from the expected 33% based on corpora counts (e.g. Brodie, 1971;Suydam, 2009), or from the cycle of one calf every three years (Vladykov, 1944; Doidge, 1990; Suydam, 2009; Mosnier *et al.,* 2015).

Our sample likely included senescent individuals. Senescence exists in beluga, with an estimated 27% of female-years being lived by post-reproductive females in a stable beluga population (Marsh and Kasuya, 1986; Ellis *et al.,* 2018). This proportion can drop to 19% in a population declining by 10% per year and might increase to 33% in a population increasing at the same rate (Ellis *et al.,* 2018). This 33% expected pregnancy rate is derived from quadratic relationships between *corpora* counts and age and generally ignores the stabilization or decline in *corpora* counts in older individuals (e.g. Suydam, 2009;Ellis *et al.,* 2018). Since our biopsy sample includes an unknown number of senescent females, our estimate of pregnancy rate for this population may be biased downwards (the denominator should be decreased by an unknown amount).

Anomalously high calf mortality, such as documented in SLE beluga since 2008 (Lesage, 2021) can increase annual pregnancy rate by making females that have lost a calf, available for mating one year earlier (Mosnier *et al.,* 2015). This is assuming that calf mortality is not consequent to poor body condition of the female, in which case a new ovulation may not arise (Lockyer, 1987; Thomas, 1990). In counterpart, inadequate food resources, like suspected for SLE beluga (Bernier-Graveline *et al.,* 2021; Lesage, 2021), may also delay reproduction and decrease pregnancy rate by lengthening calving interval (e.g. Lockyer, 1987; Thomas, 1990). There is currently no published record about reproductive history for individual SLE beluga females to estimate the relative importance, let alone the existence, of these two factors for the SLE beluga population.

A bad timing of sampling or time lag too short for the pregnancy signal to disappear from blubber may both inflate pregnancy rate. The timing of our sampling 1.5—2 mo following peak parturition should have minimized this bias. Time lag for steroid signal onset in blubber is short (1—2 h) in odontocetes (Champagne *et al.,* 2018). However, lag for its offset is unknown in cetacean or marine mammals in general (e.g. Galligan *et al.,* 2020). In the present study, only one individual in early pregnancy (2—3 mo pregnant) and none in mid-pregnancy (corresponding to the timing of our sampling) were available from SLE_carcasses_ data set: BP levels in the newly pregnant female (9.0 ng g^−1^) were higher than that for immature females or males (0.6–1.1 ng g^−1^) and at the high end of all but one resting females, suggesting a relatively rapid offset of the pregnancy signal in blubber. Lactation causes BP levels to decrease in mammals (e.g. Kuhn, 1969). Females in our carcass sample that died shortly after parturition and with still high BP levels (above 86 ng g^−1^) all lacked active lactation, suggesting a rapid decline in BP levels with lactation onset. Of the eight lactating females that died around our sampling dates (11 September—28 October), all but a very late-parturient female with high BP levels (estimated to have given birth in mid-September) had normal-size uterus, symmetrical cornu, and low BP levels (range, 0.16–85.9 ng g^−1^), again suggesting that the timing of our sampling was appropriate for a pregnancy rate generally exempt of post-partum high BP levels. However, among eight biopsied females flanked by a newborn calf, thus presumably lactating, five were correctly classified as non-pregnant or in an ‘intermediate’ cluster, but three (with BP ranging between 137 and 238 ng g^−1^) were considered pregnant. An error in field observations when associating a female with a repeatedly flanking calf cannot be ruled out—allocaring exists in SLE beluga, with alloparenting of newborn calves documented in nearly 20% of the observed events (Aubin *et al.,* 2021, 2022). Of the four females forming an exclusive pair with their calf (without other beluga), three females had levels < 30 ng g^−1^, but one female had high levels of 137 ng g^−1^. These findings combined with the observation of a late parturient among carcasses suggest that our pregnancy estimate for the free-ranging SLE beluga population might be slightly inflated by late-parturient females.

The proportion of grey individuals in our sample (15/60, or ~ 25%) was within the 22.6–39.6% range reported for this population during 1989–2012 (Michaud, 2014). However, we found unexpectedly high pregnancy rates among grey individuals (~27% versus 3% predicted), assumed to reflect the juvenile segment of our sample. However, grey colouration can persist late in adulthood in some individuals (Doidge, 1990), potentially explaining the higher-than-expected pregnancy rates for this colour class in our sample. We found no evidence in the literature that age could systematically bias progesterone quantification, although progesterone levels during pregnancy were higher in younger than older individuals in humpback whales (Lowe *et al.,* 2021). In a population below carrying capacity (K), pregnancy rate among younger adults is expected to be higher than when a population is near K (Eberhardt, 2002). Whether the high pregnancy rate for grey beluga is related to a higher production by this segment of the population resulting from the small population size is unknown. With the increased use of unoccupied aerial vehicles and aerial photogrammetry, future studies may better determine age class based on body length, thus providing fine-tuned information on age-related changes in hormonal concentrations. When it is possible, endocrine profiles could be combined with sighting histories to determine the reproductive status of individual whales (e.g. through photo-identification (Pallin *et al.,* 2018; McGuire *et al.,* 2020; Kershaw *et al.,* 2021; Melica *et al.,* 2021b)).

Our sample size being small, estimates of pregnancy rates remain sensitive to slight changes or potential errors in female assignations per reproductive or colour class. Our sampling design appeared successful at ensuring equal availability of all reproductive segments of the population. Biopsy samples were acquired remotely from a distance of 15—20 m according to range finder measurements. While avoidance behaviour might occur at those distances depending on activity and herd composition, there was no clear indication in our data of a systematic bias against mature or immature females aged 3+ years-old as they constituted respectively 82 and 18% of the sample. In this population, grey females other than newborn calves (i.e. aged 1+) were expected to represent approximately 30% of the population in 2012 (Michaud, 2014; Mosnier *et al.,* 2015).

The estimated pregnancy rate for the SLE beluga population, which is only slightly higher than the expected rate from corpora counts of one calf every three years, does not suggest a generalized incapacity of females in this population to become pregnant. None of the factors examined could have biased the pregnancy rate upwards by a large amount. Incorporating life history traits of the sampled individuals or of other recognizable individuals in the population, e.g. calving interval, age at first reproduction, age of reproductive senescence, would reduce the uncertainty around our pregnancy rate and provide essential information on lifetime reproductive success across environmental contexts. This would help to better identify impediments to recruitment, evaluate recovery scenarios and predict population growth. Given that standardization of data is a crucial element for comparison among studies, we recommend that future investigations present BP concentrations against both wet-tissue and lipid contents. We also recommend using methods with the least *a priori* information to classify individuals based on their endocrine profiles. Indeed, lipid loss in the field and carcass decomposition, among other factors, make comparisons between samples obtained through carcass and biopsy programs challenging. This study illustrates the many challenges for assessing pregnancy rates and maturity status in blindly sampled free-ranging cetaceans and the importance of a carefully designed study both in terms of timing and distribution of sampling.

## Supplementary Material

Web_Material_coad075

## Data Availability

Data are available from the Dryad Digital Repository https://doi.org/10.5061/dryad.34tmpg4r5.
