# Supplementary material for: Estimating pregnancy rate from blubber progesterone levels of a blindly biopsied beluga population poses methodological, analytical and statistical challenges
Source: Conserv Physiol. 2023 Sep 26;11(1):coad075. doi: 10.1093/conphys/coad075 (PMC10533324; doi:10.1093/conphys/coad075)
Supplement: Web_Material_coad075 [file web_material_coad075.pdf]

Estimating pregnancy rate from blubber progesterone levels of a blindly biopsied beluga population poses methodological, analytical and statistical challenges

Renaud L-A[1,1], Bordeleau X[1], Kellar NM[2], Pigeon G[3], Michaud R[4], Morin Y[1], Lair S[5], Therien A[1], Lesage V[1]

[1] Fisheries and Oceans Canada, Maurice Lamontagne Institute, P.O. Box 1000, 850 Route de la Mer, Mont-Joli, Quebec, G5H 3Z4, Canada

[2] Southwest Fisheries Science Center, National Marine Fisheries Service, P. O. Box 271, La Jolla, California 92038, USA

[3] Institut de recherche sur les forêts, Université du Québec en Abitibi-Témiscamingue, Québec, J9X 5E4, Canada

[4] Groupe de recherche et d'éducation sur les mammifères marins (GREMM), 108 de la Cale-Sèche, Tadoussac, Québec G0T 2A0, Canada

[5] Faculté de médecine vétérinaire, Université de Montréal, PO Box 5000, 3200 Rue Sicotte, St-Hyacinthe, Québec, J2S 7C6, Canada.

[1] Corresponding authors: L.-A. Renaud (limoilou-amelie.renaud@dfo-mpo.gc.ca) and V. Lesage (veronique.lesage@dfo-mpo.gc.ca), Fisheries and Oceans Canada, Maurice Lamontagne Institute, P.O. Box 1000, 850 Route de la Mer, Mont-Joli, Quebec, G5H 3Z4, Canada.

Appendix A. Supplementary methods: Sample processing and progesterone extraction

Blubber samples were homogenized in 1.2 mL of 100% ethanol using the FastPrep-24™ Classic bead beating grinder and lysis system (M.P. Biomedicals, Santa Ana, CA, USA). Blubber homogenization protocol consisted of eight 45 s cycles at a speed of 5 m/s in specialized lysing matrix A tubes (6910-500, M.P. Biomedicals, Santa Ana, CA, USA) containing an extra ceramic sphere (6540-412, M.P. Biomedicals, Santa Ana, CA, USA). The content of the lysing matrix A tube was poured into a 13 x 100 mm borosilicate culture tube. The homogenate was transferred to a new 13 x 100 mm borosilicate culture tube. An additional 1.5 mL of ethanol was used to rinse the lysing tube and the 13 x 100 mm borosilicate culture tube and added to the homogenate. Two milliliters of 4:1 ethanol:acetone was added to the homogenate. Samples were mixed for 5 min with a multi-tube vortex (Fisher Scientific, Waltham, MA, USA) and then centrifuged at 4000 rpm for 15 min. The supernatant was transferred to a 12 x 75 mm borosilicate culture tube and evaporated under compressed air with an Reacti-Vap™ III evaporation unit and Reacti-Therm™ III heating module set at 30°C. Two milliliters of diethyl ether was added to the residue, and samples were mixed and centrifuged as described above. The supernatant was transferred to a new 12 x 75 mm borosilicate culture tube and evaporated in the same conditions as above. Following evaporation, 1.5 mL of acetonitrile was added to the residue. Samples were vortexed for 5 min and then 1.5 mL of hexane was added to form 2 immiscible phases. Following another 5 min of vortexing, samples were centrifuged as above, and placed at -20°C for at least 20 min. The lower phase containing the steroids in acetonitrile was collected and transferred to a new 12 x 75 mm borosilicate culture tube. Residual lipids and steroids were re-extracted by adding 1.5 mL of hexane to the previously collected (lower) acetonitrile phase, and 1.5 mL of acetonitrile to the upper hexane layer. Samples were vortexed for 5 min, centrifuged at 4000 rpm for 15 min and placed at -20°C for at least 20

min once more. The lower acetonitrile layers of both series were combined and evaporated under compressed air as above. The final residue containing the extracted steroids was stored at -20°C until progesterone analysis. The upper hexane layers of both series were combined and transferred to a pre-weighed dish and evaporated under a flow-hood. Following hexane evaporation, the lipid residue was weighed to determine the initial lipid content of the blubber sample. Progesterone extraction efficiency was determined using spiked samples. The ethanol 100% was spiked with 0.03 ng of progesterone and subjected to the same extraction steps as above. The extraction efficiency was calculated as the percentage of the quantified amount of progesterone by the amount of progesterone added before extraction.

BP was quantified using the competitive enzyme-linked immunosorbent assay (ELISA) kit from Enzo Life Sciences (ADI-900-011). The morning of the immunoassay, duplicate samples were prepared by re-suspending the final residue from the hormone extraction in 500 µL of assay buffer (ADI-80-0010, Enzo Life Sciences, Farmingdale, NY, USA). To ensure sample concentrations would be in the accurate detection range of the assay, samples from SLE carcasses of known reproductive status were further diluted in assay buffer as follows: pregnant females were diluted 1/500; recently pregnant females were diluted 1/100 and 1/500; lactating females were diluted 1/5, 1/50, 1/100 and 1/500; mature non-pregnant females were diluted 1/5, 1/50 and 1/200; immature females were diluted 1/5; immature and mature males were diluted 1/5. Samples from animals of unknown reproductive status (i.e., $SLE_{biopsies}$ and $NUN_{carcasses}$) were each diluted 1/5, 1/100 and 1/500 in assay buffer. The assay detection limits were between 15.6 and 500 pg/mL. Samples with concentrations outside these limits were diluted further at 1/1000 and even 1/5000 (above) or were re-assayed at a lower dilution (below) to be accurately measured. Samples diluted at 1/5 that fell below the lowest standard were assayed non-diluted.

Appendix A. Supplementary results, part I: Comparisons of three statistical approaches to classify individuals based on blubber progesterone expressed in ng g^{-1} of tissue

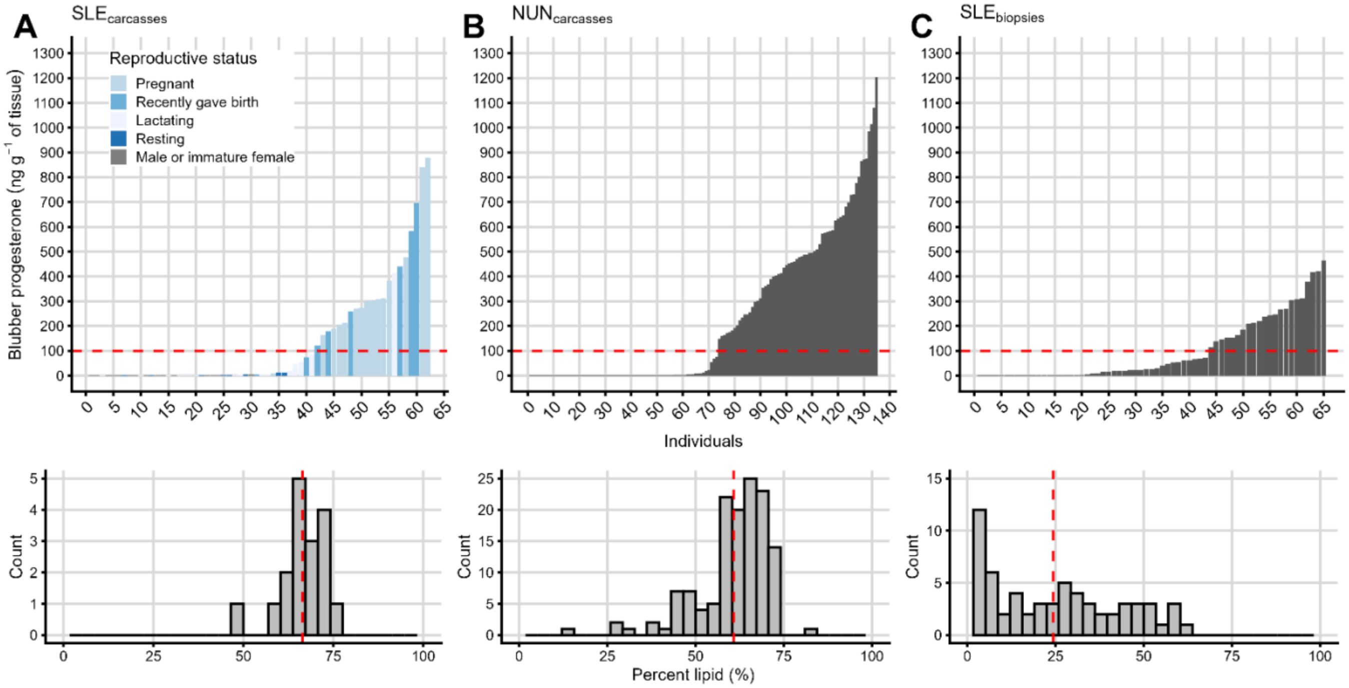

Figure A 1 Blubber progesterone concentration (ng) when reported per gram of tissue (top panel) and distribution of the lipid composition of samples (bottom panel) in A: SLE$_{carcasses}$, B: NUN$_{carcasses}$, and C: SLE$_{biopsies}$ programs. The red dashed line represents the 100 ng/g estimated threshold for pregnancy (upper panel) and mean percent lipids (bottom panel).

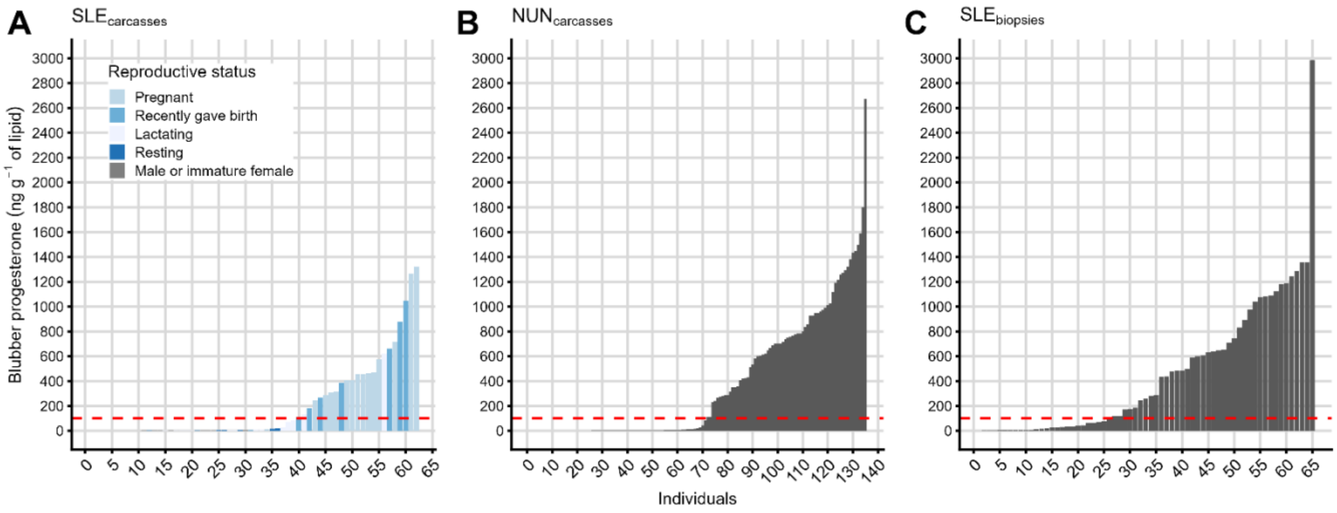

Figure A 2 Comparison of blubber progesterone (ng) when reported per gram of lipid among $SLE_{carcasses}$, $NUN_{carcasses}$, and $SLE_{biopsies}$. The red dashed line represents the 150 ng g^{-1} estimated threshold.

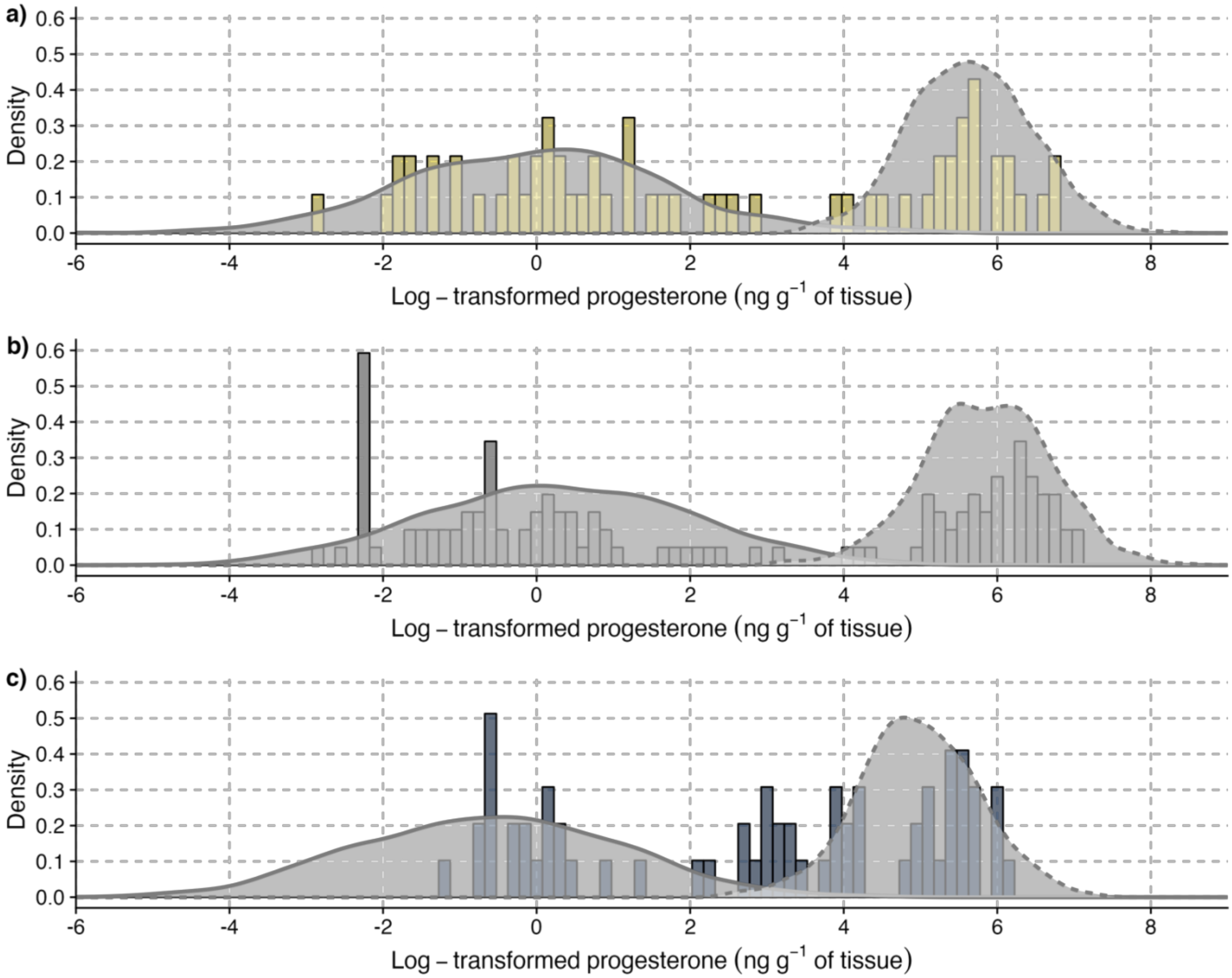

Figure A 3 Mixtures of Gaussian distributions used to assign a probability of being pregnant in belugas based on log-transformed blubber progesterone concentration. The approach is similar to Figure 3 in the main text, but it includes pooled samples from the $SLE_{carcasses}$, $NUN_{carcasses}$ and $SLE_{biopsies}$ datasets. Panel a) shows distributions for necropsied belugas from the St. Lawrence Estuary, Canada ($SLE_{carcasses}$); panel b) shows belugas harvested in Hudson Bay and Hudson Strait, Canada ($NUN_{carcasses}$); panels c) show free-ranging belugas biopsied in the St. Lawrence Estuary, Canada ($SLE_{biopsies}$). For the full description

of the different reproductive statuses observed in $SLE_{carcasses}$, refer to Figure 2 in the main text.

Histogram and density plots were put on the same scale.

Table A 1 Descriptive statistics of the Gaussian distributions of log-transformed blubber progesterone concentration (in ng g^{-1} of tissue) from the St. Lawrence Estuary ($SLE_{carcasses}$), the harvest in Hudson Bay and Hudson Strait ($NUN_{carcasses}$), and free-ranging beluga from the St. Lawrence Estuary ($SLE_{biopsies}$). A mixture of two distributions best represented log-transformed progesterone concentration in the $SLE_{carcasses,}$ and $NUN_{carcasses}$ (e.g., non-pregnant and pregnant females, Fig. A1) and three distributions for $SLE_{biopsies}$. Mean, median, standard deviation (SD) and 95% credible intervals (lower, upper) around the mean are given.

	Mean	Median	SD	95%LCI	95%UCI
**$SLE_{carcasses}$**					
Mean, presumed non-pregnant	0.2	0.2	0.3	-0.4	0.7
Mean, presumed pregnant	5.5	5.5	0.2	5.1	5.9
SD, presumed non-pregnant	1.5	1.5	0.2	1.2	2.0
SD, presumed pregnant	0.8	0.8	0.2	0.6	1.3
**$NUN_{carcasses}$**					
Mean, presumed non-pregnant	-0.3	-0.3	0.2	-0.7	0.1
Mean, presumed pregnant	6.0	6.0	0.1	5.9	6.2
SD, presumed non-pregnant	1.6	1.5	0.2	1.3	1.9
SD, presumed pregnant	0.6	0.6	0.1	0.5	0.8
**$SLE_{biopsies}$**					
Mean, presumed non-pregnant	-0.2	-0.2	0.1	-0.4	0.1
Mean, Intermediate	3.4	3.4	0.2	3.0	3.9
Mean, presumed pregnant	5.4	5.5	0.2	5.1	5.7
SD, presumed non-pregnant	0.6	0.6	0.1	0.4	0.9
SD, Intermediate	0.9	0.9	0.2	0.6	1.5
SD, presumed pregnant	0.5	0.4	0.2	0.3	0.9

Table A 2 Contingency table contrasting the effect of three statistical approaches (a fixed threshold, a mixture model, and a logistic regression) on the probability of being pregnant in three different beluga datasets, based on blubber progesterone expressed in ng g^{-1} of tissue. Missing values indicate different classifications between study programs. Samples from the $SLE_{biopsies}$ were classified into three clusters using mixture models (e.g., presumed not pregnant, Intermediate and presumed pregnant) but into two clusters (e.g., presumed not pregnant and presumed pregnant) in $NUN_{carcasses}$ and $SLE_{carcasses}$.

**Threshold**	**Cluster**	**Logistic**	**$SLE_{carcasses}$**	**$NUN_{carcasses}$**	**$SLE_{biopsies}$**
pres notPreg	pres notPreg	pres notPreg	37	71	20
pres notPreg	pres notPreg	pres preg	0	0	0
pres notPreg	pres preg	pres notPreg	2	0	0
pres notPreg	pres preg	pres preg	2	2	0
pres notPreg	pres intermediate	pres notPreg	-	-	18
pres notPreg	pres intermediate	pres preg	-	-	5
pres preg	pres notPreg	pres notPreg	0	0	0
pres preg	pres notPreg	pres preg	0	0	0
pres preg	pres preg	pres notPreg	0	0	0
pres preg	pres preg	pres preg	21	62	22
pres preg	pres intermediate	pres notPreg	-	-	0
pres preg	pres intermediate	pres preg	-	-	0

Table A 3 Classification of individuals of known ($SLE_{carcasses}$) reproductive status based on progesterone concentrations (in ng g^{-1} of tissue), made using three statistical approaches: a fixed threshold, a model-based clustering and a logistic regression. For $SLE_{carcasses}$ known reproductive status is from examination of reproductive tracts. The resulting number of individuals (N) in each category and associated mean progesterone concentrations (Mean ± standard deviation, SD) are given for each statistical approach. Classification as presumed pregnant is indicated as 'pres preg' and presumed not pregnant as 'pres notPreg'.

	100 ng g^{-1} Fixed threshold			Mixture model			Logistic		
Status from reproductive tracts	N	Mean ± SD	Classification	N	Mean ± SD	Classification	N	Mean ± SD	Classification
Pregnant									
Pregnant - dystocia	10	395 ± 250	pres preg	10	395 ± 250	pres preg	10	395 ± 250	pres preg
Early pregnancy	1	9.0	pres notPreg	1	9.0	pres notPreg	1	9.0	pres notPreg
Unspecified stage	2	392 ± 120	pres preg	2	392 ± 120	pres preg	2	392 ± 120	pres preg
Non-pregnant									
Recently gave birth	2	39.1 ± 48.5	pres notPreg	1	4.8	pres notPreg	1	4.8	pres notPreg
Recently gave birth	8	331 ± 215	pres preg	9	303 ± 219	pres preg	9	303 ± 219	pres preg
Lactating	16	15.0 ± 25.3	pres notPreg	13	3.9 ± 5.0	pres notPreg	15	10.2 ± 17.3	pres notPreg
Lactating	1	413	pres preg	4	150 ± 176	pres preg	2	249 ± 231	pres preg
Resting	7	3.1 ± 4.0	pres notPreg	7	3.1 ± 4.0	pres notPreg	7	3.1 ± 4.0	pres notPreg
Male or immature female	15	0.8 ± 0.9	pres notPreg	15	0.8 ± 0.9	pres notPreg	15	0.8 ± 0.9	pres notPreg

Table A 4 Classification of individuals of known ($SLE_{carcasses}$) and unknown ($NUN_{carcasses}$ and $SLE_{biopsies}$) reproductive status based on progesterone concentrations (in ng g^{-1} of tissue). The three statistical approaches were similar to those presented in Table 2 of the main text, except that samples were pooled across datasets. For $SLE_{carcasses}$ the known number of individuals in each category is indicated in parenthesis. The resulting number of individuals (N) in each category and associated mean progesterone concentrations (Mean ± standard deviation, SD) are given for each statistical approach.

	100 ng g^{-1} Fixed threshold		Mixture model		Logistic	
	N	Mean ± SD	N	Mean ± SD	N	Mean ± SD
**$SLE_{carcasses}$**						
Presumed non-pregnant (49)	41	8.8 ± 19.8	37	2.7 ± 3.8	39	5.2 ± 11.5
Presumed pregnant (13)	21	371 ± 215	25	323 ± 227	23	346 ± 222
**$NUN_{carcasses}$**						
Presumed non-pregnant	73	4.5 ± 13.4	70	1.9 ± 3.9	71	2.6 ± 7.2
Presumed pregnant	62	497 ± 245	65	477 ± 256	64	483 ± 253
**$SLE_{biopsies\ (All\ samples\ included)}$**						
Presumed non-pregnant	43	18.4 ± 22.6	27	3.9 ± 5.8	40	14.7 ± 18.6
Presumed pregnant	22	253 ± 99	38	164 ± 129	25	231 ± 111

Supplementary results, part II: Comparisons of three statistical approaches to classify individuals based on blubber progesterone expressed in ng g^{-1} of lipid.

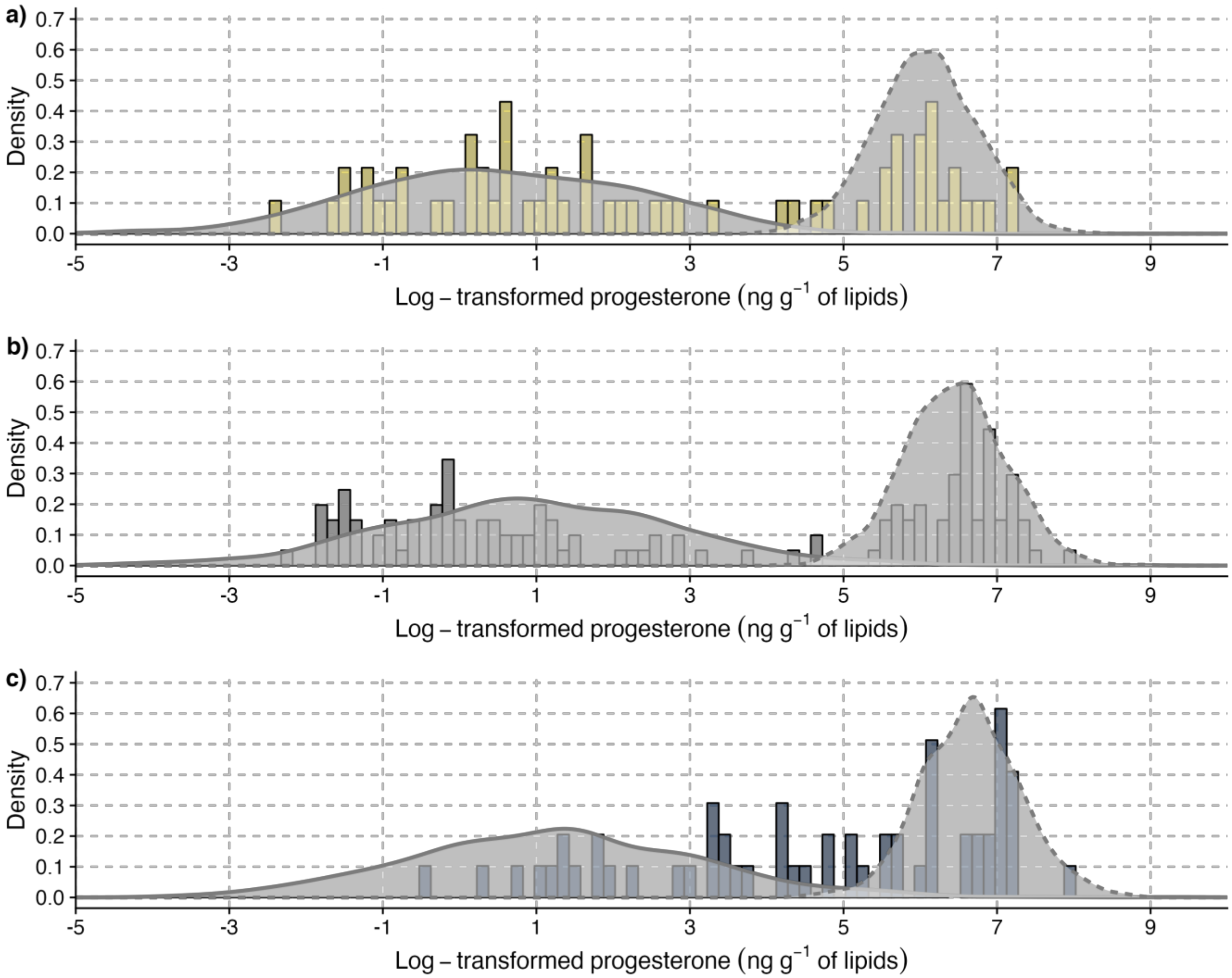

Figure A 4 Mixtures of Gaussian distributions used to assign a probability of being pregnant in belugas based on log-transformed blubber progesterone concentration. The approach is similar to Figure A. 3. with progesterone concentration corrected for sample lipid content (in ng g^{-1} of lipids). Panel a) shows distributions for $SLE_{carcasses}$ belugas from the St. Lawrence Estuary, Canada; panel b) shows $NUN_{carcasses}$ harvested in Hudson Bay and Hudson Strait, Canada; panel c) shows free-ranging beluga biopsied in the

St. Lawrence Estuary, Canada ($SLE_{biopsies}$). For the full description of the different reproductive statuses observed in $SLE_{carcasses}$, refer to Figure 2 in the main text.

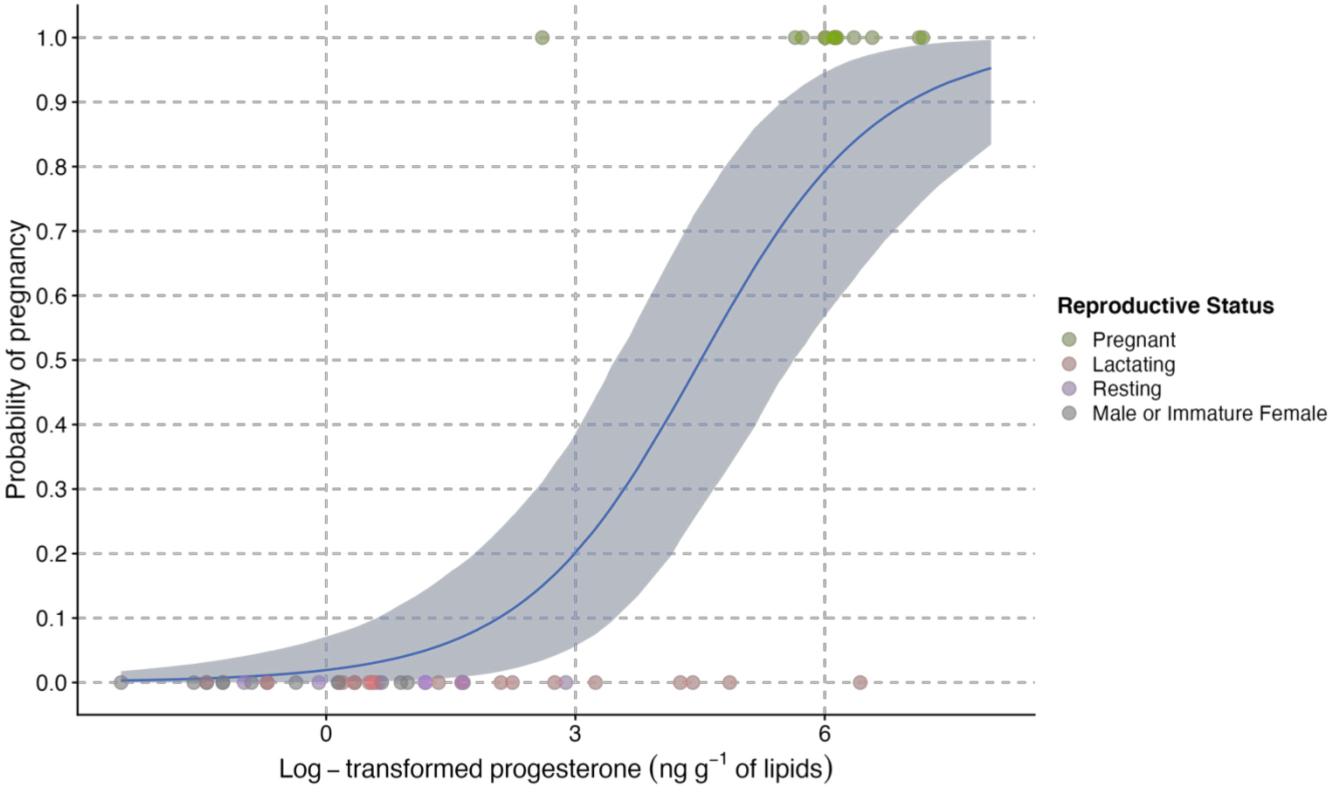

Figure A 5 Logistic regression was used to predict the probability of being pregnant in beluga based on their blubber progesterone concentration expressed in ng g^{-1} of lipids. The GLM used individuals of known reproductive status as input data (here, carcasses from the $SLE_{carcasses}$ dataset). Individuals of unknown reproductive status (here, the $SLE_{biopsies}$) were predicted by the GLM.

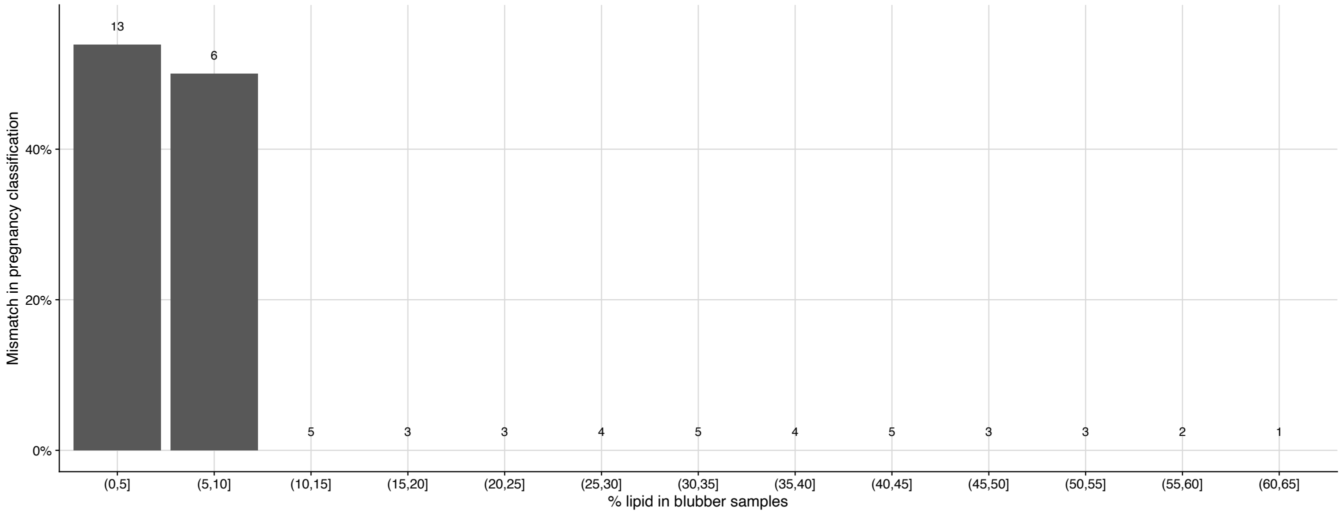

Figure A 6 The effect of correcting for sample lipid content (% of sample weight) on the classification of individual samples based on their blubber progesterone concentration and a model-based clustering. When progesterone is corrected for lipid content in the sample, 10 samples change from the ‘low’ to the ‘high’ progesterone cluster, and are thus ‘mismatched’. Of the 13 samples in the 0 – 5% lipid class, 7 (53.4%) change from the ‘low’ to ‘high’ cluster, and 3/6 (50%) of samples in the 5 – 10% lipid class change from the ‘low’ to ‘high’ cluster. ‘(’ and ‘]’ indicates that the right interval is closed, and the left is open.

Table A 5 Decriptive statistics of the Gaussian distributions of log-transformed blubber progesterone concentration (in ng g^{-1} of lipids) from the St. Lawrence Estuary ($SLE_{carcasses}$), the harvest in Hudson Bay and Hudson Strait ($NUN_{carcasses}$), and free-ranging beluga from the St. Lawrence Estuary ($SLE_{biopsies}$). A mixture of two distributions best represented log-transformed progesterone concentration in all datasets (e.g., non-pregnant and pregnant females; Fig. A3). Mean, median, standard deviation (SD) and 95% credible intervals (lower, upper) around the mean are given.

	**Mean**	**Median**	**SD**	**95%LCI**	**95%UCI**
**$SLE_{carcasses}$**					
Mean, presumed non-pregnant	0.6	0.6	0.3	0.1	1.1
Mean, presumed pregnant	5.9	5.9	0.2	5.5	6.3
SD, presumed non-pregnant	1.5	1.5	0.2	1.2	2.0
SD, presumed pregnant	0.8	0.8	0.2	0.6	1.2
**$NUN_{carcasses}$**					
Mean, presumed non-pregnant	0.2	0.2	0.2	-0.2	0.6
Mean, presumed pregnant	6.5	6.5	0.1	6.3	6.7
SD, presumed non-pregnant	1.5	1.5	0.2	1.3	1.9
SD, presumed pregnant	0.6	0.6	0.1	0.5	0.8
**$SLE_{biopsies}$**					
Mean, presumed non-pregnant	3.3	3.3	0.4	2.5	4.1
Mean, presumed pregnant	6.6	6.6	0.1	6.3	6.8
SD, presumed non-pregnant	1.9	1.9	0.3	1.4	2.5
SD, presumed pregnant	0.6	0.6	0.1	0.3	0.9

Table A 6 Contingency table contrasting the effect of three statistical approaches (a fixed threshold, a mixture model, and a logistic regression) on the probability of being pregnant in three different beluga datasets, based on blubber progesterone expressed in ng g^{-1} of lipids. Samples from the $SLE_{biopsies}$, $NUN_{carcasses}$, and $SLE_{carcasses}$ were classified into two clusters using mixture models (e.g., presumed not pregnant and presumed pregnant).

**Threshold**	**Cluster**	**Logistic**	**$SLE_{carcasses}$**	**$NUN_{carcasses}$**	**$SLE_{biopsies}$**
pres notPreg	pres notPreg	pres notPreg	37	71	26
pres notPreg	pres notPreg	pres preg	0	0	2
pres notPreg	pres preg	pres notPreg	2	0	0
pres notPreg	pres preg	pres preg	2	2	0
pres preg	pres notPreg	pres notPreg	0	0	0
pres preg	pres notPreg	pres preg	0	0	5
pres preg	pres preg	pres notPreg	0	0	0
pres preg	pres preg	pres preg	21	62	32

Table A 7 Classification of individuals of known ($SLE_{carcasses}$) and unknown ($NUN_{carcasses}$ and $SLE_{biopsies}$) reproductive status using similar approaches as in Table 2, but progesterone concentrations expressed in ng per gram of lipid and samples were pooled across datasets. For $SLE_{carcasses}$ the known number of individuals in each category is indicated in parenthesis. The resulting number of individuals (N) in each category and associated mean progesterone concentrations (Mean ± standard deviation, SD) are given for each statistical approach.

	150 ng g^{-1} Fixed threshold		Mixture model		Logistic	
	N	Mean ± SD	N	Mean ± SD	N	Mean ± SD
**$SLE_{carcasses}$**						
Presumed non-pregnant (49)	41	13.2 ± 29.6	38	5.8 ± 12.2	39	7.7 ± 17.2
Presumed pregnant (13)	21	556 ± 322	24	500 ± 336	23	518 ± 332
**$NUN_{carcasses}$**						
Presumed non-pregnant	73	7.3 ± 20.8	73	7.3 ± 20.8	71	4.4 ± 11.3
Presumed pregnant	62	820 ± 448	62	820 ± 448	64	798 ± 458
**$SLE_{biopsies\ (All\ samples\ included)}$**						
Presumed non-pregnant	28	32.5 ± 35.3	28	32.5 ± 35.3	26	25.9 ± 26.6
Presumed pregnant	37	782 ± 520	37	782 ± 520	39	748 ± 527

Appendix B. Supplementary results, part I: Individual probabilities of pregnancy as estimated by three approaches, based on blubber progesterone expressed in ng g^{-1} of tissue.

Table B 1 Estimated probability of being pregnant for St. Lawrence Estuary beluga necropsied between 1997 and 2019, Quebec, Canada. Three statistical approaches were compared to assign a probability of being pregnant: a 100-ng g^{-1} threshold, a model-based clustering and a logistic regression of progesterone concentrations (ng g^{-1} of tissue). For each animal, it is reported sex, age, progesterone concentrations (ng g^{-1} of tissue), known reproductive status based on examination of reproductive tracts, sexual maturity (mature, immature), and reproductive status based on model classification for each statistical approach. Bold characters indicate inaccurate classification.

Sample	Sex	Age	Sampling date	Progesterone	Reproductive status	Maturity	100 ng g^{-1} threshold	Mixture model			Logistic regression		
				(ng g^{-1} tissue)			Classification	Prob. notPreg	Prob. pregnant	Classification	Mean Prob.	95% CI	Classification
DL1997-002	F	57	1997-05-23	1.1	lactating	M	pres notPreg	1.0	0.0	pres notPreg	0.0	0-0.1	pres notPreg
DL1997-003	F	43	1997-06-08	477	pregnant	M	pres preg	0.0	1.0	pres preg	0.9	0.7-1	pres preg
DL1997-005	M	12	1997-07-15	1.6	-	I	pres notPreg	1.0	0.0	pres notPreg	0.0	0-0.1	pres notPreg
DL1997-006	F	62	1997-07-29	0.2	resting	M	pres notPreg	1.0	0.0	pres notPreg	0.0	0-0	pres notPreg
DL1998-003	F	59	1998-05-24	11.9	resting	M	pres notPreg	1.0	0.0	pres notPreg	0.2	0.1-0.4	pres notPreg
DL1998-004	F	42	1998-05-24	1.3	resting	M	pres notPreg	1.0	0.0	pres notPreg	0.0	0-0.1	pres notPreg
DL1998-007	M	36	1998-08-07	0.9	-	M	pres notPreg	1.0	0.0	pres notPreg	0.0	0-0.1	pres notPreg
DL1999-009	F	39	1999-10-15	2.2	resting	M	pres notPreg	1.0	0.0	pres notPreg	0.1	0-0.1	pres notPreg
DL2000-004	F	-	2000-10-06	3.4	lactating	M	pres notPreg	1.0	0.0	pres notPreg	0.1	0-0.2	pres notPreg
DL2000-005	F	50	2000-10-12	10.4	lactating	M	pres notPreg	1.0	0.0	pres notPreg	0.2	0-0.3	pres notPreg
DL2001-005	M	7	2001-09-25	3.5	-	I	pres notPreg	1.0	0.0	pres notPreg	0.1	0-0.2	pres notPreg
DL2002-005	F	27	2002-07-25	206	pregnant	M	pres preg	0.0	1.0	pres preg	0.8	0.5-0.9	pres preg
DL2003-002	F	45	2003-06-13	9.0	pregnant	M	**pres notPreg**	**1.0**	**0.0**	**pres notPreg**	**0.2**	**0-0.3**	**pres notPreg**
DL2003-006	F	52	2003-10-17	3.4	resting	M	pres notPreg	1.0	0.0	pres notPreg	0.1	0-0.2	pres notPreg
DL2003-007	F	30	2003-10-22	0.2	lactating	M	pres notPreg	1.0	0.0	pres notPreg	0.0	0-0	pres notPreg
DL2004-003	F	56	2004-06-11	0.8	lactating	M	pres notPreg	1.0	0.0	pres notPreg	0.0	0-0.1	pres notPreg
DL2004-004	M	3	2004-06-19	0.2	-	I	pres notPreg	1.0	0.0	pres notPreg	0.0	0-0	pres notPreg
DL2005-004	M	50	2005-09-04	0.2	-	M	pres notPreg	1.0	0.0	pres notPreg	0.0	0-0	pres notPreg
DL2006-002	F	4	2006-09-17	0.3	-	I	pres notPreg	1.0	0.0	pres notPreg	0.0	0-0	pres notPreg
DL2007-002	F	44	2007-05-27	0.9	lactating	M	pres notPreg	1.0	0.0	pres notPreg	0.0	0-0.1	pres notPreg
DL2007-004	F	28	2007-07-03	211	recently gave birth	M	pres preg	0.0	1.0	pres preg	0.8	0.5-0.9	pres preg

DL2007-008	F	38	2007-09-11	0.3	lactating	M	pres notPreg	1.0	0.0	pres notPreg	0.0	0-0	pres notPreg
DL2007-009	M	50	2007-09-22	1.8	-	M	pres notPreg	1.0	0.0	pres notPreg	0.0	0-0.1	pres notPreg
DL2008-003	F	52	2008-06-16	5.5	lactating	M	pres notPreg	1.0	0.0	pres notPreg	0.1	0-0.2	pres notPreg
DL2008-007	F	57	2008-09-18	1.2	lactating	M	pres notPreg	1.0	0.0	pres notPreg	0.0	0-0.1	pres notPreg
DL2009-001	F	12	2009-06-16	413	lactating	M	**pres preg**	**0.0**	**1.0**	**pres preg**	**0.8**	**0.6-1**	**pres preg**
DL2010-001	F	2	2010-03-09	0.3	-	I	pres notPreg	1.0	0.0	pres notPreg	0.0	0-0	pres notPreg
DL2010-002	F	1	2010-03-21	0.5	-	I	pres notPreg	1.0	0.0	pres notPreg	0.0	0-0.1	pres notPreg
DL2010-005	F	51	2010-06-10	307	pregnant	M	pres preg	0.0	1.0	pres preg	0.8	0.6-1	pres preg
DL2010-006	F	18	2010-07-16	179	recently gave birth	M	pres preg	0.0	1.0	pres preg	0.7	0.5-0.9	pres preg
DL2010-007	F	40	2010-07-31	383	pregnant	M	pres preg	0.0	1.0	pres preg	0.8	0.6-1	pres preg
DL2010-008	F	31	2010-08-29	189	pregnant	M	pres preg	0.0	1.0	pres preg	0.7	0.5-0.9	pres preg
DL2010-011	F	35	2010-12-31	2.2	resting	M	pres notPreg	1.0	0.0	pres notPreg	0.1	0-0.1	pres notPreg
DL2011-001	M	2	2011-04-07	0.8	-	I	pres notPreg	1.0	0.0	pres notPreg	0.0	0-0.1	pres notPreg
DL2011-002	F	44	2011-05-19	840	pregnant	M	pres preg	0.0	1.0	pres preg	0.9	0.7-1	pres preg
DL2011-003	F	30	2011-06-08	878	pregnant	M	pres preg	0.0	1.0	pres preg	0.9	0.7-1	pres preg
DL2011-004	F	56	2011-07-12	17.1	lactating	M	pres notPreg	0.9	0.1	pres notPreg	0.2	0.1-0.4	pres notPreg
DL2011-005	F	14	2011-08-09	271	pregnant	M	pres preg	0.0	1.0	pres preg	0.8	0.6-1	pres preg
DL2011-006	F	68	2011-09-10	0.6	resting	M	pres notPreg	1.0	0.0	pres notPreg	0.0	0-0.1	pres notPreg
DL2011-008	F	22	2011-10-28	85.9	lactating	M	pres notPreg	**0.0**	**1.0**	**pres preg**	**0.6**	**0.3-0.8**	**pres preg**
DL2011-009	M	43	2011-11-09	0.1	-	M	pres notPreg	1.0	0.0	pres notPreg	0.0	0-0	pres notPreg
DL2012-001	F	26	2012-05-19	73.4	recently gave birth	M	**pres notPreg**	0.1	0.9	pres preg	0.5	0.3-0.8	pres preg
DL2012-003	F	21	2012-07-17	440	recently gave birth	M	pres preg	0.0	1.0	pres preg	0.9	0.6-1	pres preg
DL2013-005	M	21	2013-09-15	0.8	-	M	pres notPreg	1.0	0.0	pres notPreg	0.0	0-0.1	pres notPreg
DL2014-003	F	32	2014-09-28	55.1	lactating	M	pres notPreg	**0.2**	**0.8**	**pres preg**	0.5	0.2-0.7	pres notPreg
DL2015-002	M	6	2015-05-31	0.1	-	I	pres notPreg	1.0	0.0	pres notPreg	0.0	0-0	pres notPreg
DL2015-003	F	21	2015-07-17	302	pregnant	M	pres preg	0.0	1.0	pres preg	0.8	0.6-1	pres preg
DL2015-006	F	33	2015-08-18	163	recently gave birth	M	pres preg	0.0	1.0	pres preg	0.7	0.5-0.9	pres preg
DL2017-001	F	53	2017-05-20	6.3	lactating	M	pres notPreg	1.0	0.0	pres notPreg	0.1	0-0.3	pres notPreg
DL2017-003	F	43	2017-09-20	120	recently gave birth	M	pres preg	0.0	1.0	pres preg	0.7	0.4-0.9	pres preg
DL2017-004	F	45	2017-10-23	47.4	lactating	M	pres notPreg	**0.3**	**0.7**	**pres preg**	0.4	0.2-0.7	pres notPreg
DL2018-001	F	47	2018-05-20	1.2	lactating	M	pres notPreg	1.0	0.0	pres notPreg	0.0	0-0.1	pres notPreg
DL2018-002	F	18	2018-07-15	257	recently gave birth	M	pres preg	0.0	1.0	pres preg	0.8	0.5-0.9	pres preg
DL2018-003	F	36	2018-07-22	583	recently gave birth	M	pres preg	0.0	1.0	pres preg	0.9	0.7-1	pres preg
DL2018-004	F	45	2018-07-29	2.6	lactating	M	pres notPreg	1.0	0.0	pres notPreg	0.1	0-0.2	pres notPreg

DL2019-001	F	5	2019-04-08	1.3	-	I	pres notPreg	1.0	0.0	pres notPreg	0.0	0-0.1	pres notPreg
DL2019-002	M	2	2019-05-13	0.2	-	I	pres notPreg	1.0	0.0	pres notPreg	0.0	0-0	pres notPreg
DL2019-004	F	33	2019-07-04	312	pregnant	M	pres preg	0.0	1.0	pres preg	0.8	0.6-1	pres preg
DL2019-005	F	22	2019-07-16	271	pregnant	M	pres preg	0.0	1.0	pres preg	0.8	0.6-1	pres preg
DL2019-006	F	48	2019-08-01	4.8	recently gave birth	M	**pres notPreg**	**1.0**	**0.0**	**pres notPreg**	**0.1**	**0-0.2**	**pres notPreg**
DL2019-008	F	30	2019-10-17	695	recently gave birth	M	pres preg	0.0	1.0	pres preg	0.9	0.7-1	pres preg
DL2019-009	F	28	2019-11-09	303	pregnant	M	pres preg	0.0	1.0	pres preg	0.8	0.6-1	pres preg

Table B 2 Estimated probability of being pregnant for Nunavik beluga whales hunted between 2001 and 2019, Quebec, Canada. Three statistical approaches were compared to assign a probability of being pregnant: a 100-ng g^{-1} threshold, a model-based clustering and a logistic regression of progesterone concentrations (ng g^{-1} of tissue). For each animal, it is reported progesterone concentrations (ng g^{-1} of tissue), probability of a whale to be non-pregnant or pregnant, and reproductive status based on model classification for each statistical approach. All samples were from females and were of unknown reproductive status. Bold characters indicate different classifications.

Sample	Sampling date	Progesterone	100 ng g^{-1} threshold	Mixture model			Logistic regression		
		(ng g^{-1} tissue)	Classification	Prob. notPreg	Prob. pregnant	Classification	Mean Prob.	95% CI	Classification
DL-10001	2008-06-09	495	pres preg	0.0	1.0	pres preg	0.9	0.7–1	pres preg
DL-10002	2008-06-11	494	pres preg	0.0	1.0	pres preg	0.9	0.7–1	pres preg
DL-10004	2008-06-15	0.6	pres notPreg	1.0	0.0	pres notPreg	0.0	0–0.1	pres notPreg
DL-10008	2008-06-10	729	pres preg	0.0	1.0	pres preg	0.9	0.7–1	pres preg
DL-10012	2008-06-10	160	pres preg	0.0	1.0	pres preg	0.7	0.5–0.9	pres preg
DL-10014	2008-06-15	0.2	pres notPreg	1.0	0.0	pres notPreg	0.0	0–0	pres notPreg
DL-10102	2008-06-19	1.4	pres notPreg	1.0	0.0	pres notPreg	0.0	0–0.1	pres notPreg
DL-10103	2008-06-23	645	pres preg	0.0	1.0	pres preg	0.9	0.7–1	pres preg
DL-10105	2008-06-19	172	pres preg	0.0	1.0	pres preg	0.7	0.5–0.9	pres preg
DL-8047	2016-11-10	0.4	pres notPreg	1.0	0.0	pres notPreg	0.0	0–0.1	pres notPreg
DL-9076	2007-09-19	1.6	pres notPreg	1.0	0.0	pres notPreg	0.0	0–0.1	pres notPreg
DL-9129	2007-06-29	2.1	pres notPreg	1.0	0.0	pres notPreg	0.0	0–0.1	pres notPreg
DL-9140	2007-06-27	2.1	pres notPreg	1.0	0.0	pres notPreg	0.0	0–0.2	pres notPreg
DL-9143	2007-06-24	584	pres preg	0.0	1.0	pres preg	0.9	0.7–1	pres preg
DL-9156	2007-06-22	7.1	pres notPreg	1.0	0.0	pres notPreg	0.1	0–0.3	pres notPreg
DL-9164	2010-06-15	176	pres preg	0.0	1.0	pres preg	0.7	0.5–0.9	pres preg
DL-9206	2009-10-11	234	pres preg	0.0	1.0	pres preg	0.8	0.6–0.9	pres preg
DL-9215	2007-07-21	162	pres preg	0.0	1.0	pres preg	0.7	0.5–0.9	pres preg
DL-9217	2007-07-22	0.1	pres notPreg	1.0	0.0	pres notPreg	0.0	0–0	pres notPreg
DL09-174	2009-07-25	193	pres preg	0.0	1.0	pres preg	0.8	0.5–0.9	pres preg
DL09-183	2009-07-07	201	pres preg	0.0	1.0	pres preg	0.8	0.5–0.9	pres preg
DL09-212	2009-06-26	0.1	pres notPreg	1.0	0.0	pres notPreg	0.0	0–0	pres notPreg
DL09-27	2009-10-17	575	pres preg	0.0	1.0	pres preg	0.9	0.7–1	pres preg
DL09-71	2009-06-21	0.4	pres notPreg	1.0	0.0	pres notPreg	0.0	0–0.1	pres notPreg
DL09-74	2009-06-20	0.4	pres notPreg	1.0	0.0	pres notPreg	0.0	0–0.1	pres notPreg
DL09-81	-	53.3	pres notPreg	0.6	0.4	pres notPreg	0.5	0.3–0.7	pres notPreg
DL09-86	2009-06-24	2.3	pres notPreg	1.0	0.0	pres notPreg	0.1	0–0.2	pres notPreg

DL09-88	2009-06-27	1.1	pres notPreg	1.0	0.0	pres notPreg	0.0	0–0.1	pres notPreg
DL10-134	2010-10-12	1.0	pres notPreg	1.0	0.0	pres notPreg	0.0	0–0.1	pres notPreg
DL10-169	2010-11-06	509	pres preg	0.0	1.0	pres preg	0.9	0.7–1	pres preg
DL10-173	2010-06-29	453	pres preg	0.0	1.0	pres preg	0.9	0.7–1	pres preg
DL10-202	2010-06-17	1.2	pres notPreg	1.0	0.0	pres notPreg	0.0	0–0.1	pres notPreg
DL10-24	2012-06-17	412	pres preg	0.0	1.0	pres preg	0.9	0.6–1	pres preg
DL10-242	2012-07-01	1203	pres preg	0.0	1.0	pres preg	0.9	0.8–1	pres preg
DL10-8	2010-06-12	246	pres preg	0.0	1.0	pres preg	0.8	0.6–0.9	pres preg
DL12-053	2012-06-17	1.6	pres notPreg	1.0	0.0	pres notPreg	0.0	0–0.1	pres notPreg
DL12-154	2012-06-11	732	pres preg	0.0	1.0	pres preg	0.9	0.7–1	pres preg
DL12-157	2012-06-12	0.1	pres notPreg	1.0	0.0	pres notPreg	0.0	0–0	pres notPreg
DL12-169	2012-06-11	456	pres preg	0.0	1.0	pres preg	0.9	0.7–1	pres preg
DL12-273	2012-06-05	446	pres preg	0.0	1.0	pres preg	0.9	0.7–1	pres preg
DL12-292	2012-10-23	626	pres preg	0.0	1.0	pres preg	0.9	0.7–1	pres preg
DL12-293	2012-10-22	354	pres preg	0.0	1.0	pres preg	0.8	0.6–1	pres preg
DL13-020	2018-07-20	2.1	pres notPreg	1.0	0.0	pres notPreg	0.0	0–0.1	pres notPreg
DL13-026	2015-06-22	0.4	pres notPreg	1.0	0.0	pres notPreg	0.0	0–0.1	pres notPreg
DL13-055	2013-06-23	680	pres preg	0.0	1.0	pres preg	0.9	0.7–1	pres preg
DL13-085	2013-06-22	640	pres preg	0.0	1.0	pres preg	0.9	0.7–1	pres preg
DL13-096	2014-06-23	0.1	pres notPreg	1.0	0.0	pres notPreg	0.0	0–0	pres notPreg
DL13-097	2014-06-23	0.1	pres notPreg	1.0	0.0	pres notPreg	0.0	0–0	pres notPreg
DL13-099	2014-06-23	11.1	pres notPreg	1.0	0.0	pres notPreg	0.2	0–0.4	pres notPreg
DL13-109	2014-06-23	221	pres preg	0.0	1.0	pres preg	0.8	0.5–0.9	pres preg
DL13-114	2014-06-26	23.8	pres notPreg	1.0	0.0	pres notPreg	0.3	0.1–0.5	pres notPreg
DL13-116	2015-11-04	255	pres preg	0.0	1.0	pres preg	0.8	0.6–0.9	pres preg
DL13-117	2015-11-04	529	pres preg	0.0	1.0	pres preg	0.9	0.7–1	pres preg
DL13-118	2015-11-04	0.1	pres notPreg	1.0	0.0	pres notPreg	0.0	0–0	pres notPreg
DL13-125	2014-10-29	0.1	pres notPreg	1.0	0.0	pres notPreg	0.0	0–0	pres notPreg
DL13-130	2014-10-29	1.8	pres notPreg	1.0	0.0	pres notPreg	0.0	0–0.1	pres notPreg
DL13-132	2013-07-24	0.2	pres notPreg	1.0	0.0	pres notPreg	0.0	0–0	pres notPreg
DL13-182	2013-06-28	398	pres preg	0.0	1.0	pres preg	0.9	0.6–1	pres preg
DL13-185	2013-06-28	0.6	pres notPreg	1.0	0.0	pres notPreg	0.0	0–0.1	pres notPreg
DL13-191	2013-06-28	0.7	pres notPreg	1.0	0.0	pres notPreg	0.0	0–0.1	pres notPreg
DL13-192	2013-06-28	0.3	pres notPreg	1.0	0.0	pres notPreg	0.0	0–0.1	pres notPreg
DL13-241	2019-10-25	502	pres preg	0.0	1.0	pres preg	0.9	0.7–1	pres preg

DL13-sani-05	2013-06-07	1.4	pres notPreg	1.0	0.0	pres notPreg	0.0	0–0.1	pres notPreg
DL14-082	2014-06-17	488	pres preg	0.0	1.0	pres preg	0.9	0.7–1	pres preg
DL14-179	2014-06-12	1.6	pres notPreg	1.0	0.0	pres notPreg	0.0	0–0.1	pres notPreg
DL14-302	2014-08-18	0.4	pres notPreg	1.0	0.0	pres notPreg	0.0	0–0.1	pres notPreg
DL15-029	2015-06-27	0.2	pres notPreg	1.0	0.0	pres notPreg	0.0	0–0	pres notPreg
DL15-037	2015-06-23	147	pres preg	0.0	1.0	pres preg	0.7	0.5–0.9	pres preg
DL15-042	2015-06-23	408	pres preg	0.0	1.0	pres preg	0.9	0.6–1	pres preg
DL15-113	2015-10-28	367	pres preg	0.0	1.0	pres preg	0.8	0.6–1	pres preg
DL15-120	2015-06-26	0.6	pres notPreg	1.0	0.0	pres notPreg	0.0	0–0.1	pres notPreg
DL15-129	2015-07-07	0.4	pres notPreg	1.0	0.0	pres notPreg	0.0	0–0.1	pres notPreg
DL15-159	2015-10-28	6.4	pres notPreg	1.0	0.0	pres notPreg	0.1	0–0.3	pres notPreg
DL15-185	2017-06-17	874	pres preg	0.0	1.0	pres preg	0.9	0.7–1	pres preg
DL15-187	2017-06-17	0.2	pres notPreg	1.0	0.0	pres notPreg	0.0	0–0	pres notPreg
DL15-188	2017-06-17	579	pres preg	0.0	1.0	pres preg	0.9	0.7–1	pres preg
DL15-193	2018-10-17	0.3	pres notPreg	1.0	0.0	pres notPreg	0.0	0–0.1	pres notPreg
DL15-197	-	486	pres preg	0.0	1.0	pres preg	0.9	0.7–1	pres preg
DL15-244	-	68.0	pres notPreg	0.4	0.6	pres preg	0.5	0.3–0.8	pres preg
DL15-246	-	3.0	pres notPreg	1.0	0.0	pres notPreg	0.1	0–0.2	pres notPreg
DL15-249	-	697	pres preg	0.0	1.0	pres preg	0.9	0.7–1	pres preg
DL15-250	-	0.5	pres notPreg	1.0	0.0	pres notPreg	0.0	0–0.1	pres notPreg
DL15-252	-	403	pres preg	0.0	1.0	pres preg	0.9	0.6–1	pres preg
DL15-266	2015-07-23	0.2	pres notPreg	1.0	0.0	pres notPreg	0.0	0–0	pres notPreg
DL15-Quaqtaq1	2015-11-09	310	pres preg	0.0	1.0	pres preg	0.8	0.6–1	pres preg
DL16-013	2016-07-09	800	pres preg	0.0	1.0	pres preg	0.9	0.7–1	pres preg
DL16-017	2016-07-09	476	pres preg	0.0	1.0	pres preg	0.9	0.7–1	pres preg
DL16-105	2016-11-11	0.1	pres notPreg	1.0	0.0	pres notPreg	0.0	0–0	pres notPreg
DL16-118	2016-06-22	482	pres preg	0.0	1.0	pres preg	0.9	0.7–1	pres preg
DL16-137	2016-06-30	298	pres preg	0.0	1.0	pres preg	0.8	0.6–1	pres preg
DL16-140	2016-07-09	0.6	pres notPreg	1.0	0.0	pres notPreg	0.0	0–0.1	pres notPreg
DL16-144	2017-06-30	1.0	pres notPreg	1.0	0.0	pres notPreg	0.0	0–0.1	pres notPreg
DL16-279	2016-07-28	0.1	pres notPreg	1.0	0.0	pres notPreg	0.0	0–0	pres notPreg
DL16-289	2016-07-05	300	pres preg	0.0	1.0	pres preg	0.8	0.6–1	pres preg
DL17-063	2019-06-27	182	pres preg	0.0	1.0	pres preg	0.7	0.5–0.9	pres preg
DL17-114	2017-10-08	1.3	pres notPreg	1.0	0.0	pres notPreg	0.0	0–0.1	pres notPreg
DL17-123	2017-06-26	586	pres preg	0.0	1.0	pres preg	0.9	0.7–1	pres preg

DL17-125	2017-07-03	870	pres preg	0.0	1.0	pres preg	0.9	0.7–1	pres preg
DL17-154	2017-06-28	0.9	pres notPreg	1.0	0.0	pres notPreg	0.0	0–0.1	pres notPreg
DL17-232	2018-06-23	246	pres preg	0.0	1.0	pres preg	0.8	0.6–0.9	pres preg
DL17-244	2017-06-22	9.1	pres notPreg	1.0	0.0	pres notPreg	0.2	0–0.3	pres notPreg
DL17-315	2017-07-08	2.4	pres notPreg	1.0	0.0	pres notPreg	0.1	0–0.2	pres notPreg
DL17-321	2017-07-24	361	pres preg	0.0	1.0	pres preg	0.8	0.6–1	pres preg
DL17-339	2017-06-27	8.1	pres notPreg	1.0	0.0	pres notPreg	0.1	0–0.3	pres notPreg
DL17-366	2019-06-15	469	pres preg	0.0	1.0	pres preg	0.9	0.7–1	pres preg
DL18-110	2018-07-10	864	pres preg	0.0	1.0	pres preg	0.9	0.7–1	pres preg
DL18-132	2018-07-15	1014	pres preg	0.0	1.0	pres preg	0.9	0.8–1	pres preg
DL18-148	2018-07-11	459	pres preg	0.0	1.0	pres preg	0.9	0.7–1	pres preg
DL18-179	2018-07-23	0.1	pres notPreg	1.0	0.0	pres notPreg	0.0	0–0	pres notPreg
DL18-186	2018-10-29	775	pres preg	0.0	1.0	pres preg	0.9	0.7–1	pres preg
DL18-214	2018-10-16	0.3	pres notPreg	1.0	0.0	pres notPreg	0.0	0–0.1	pres notPreg
DL18-268	2018-07-07	0.1	pres notPreg	1.0	0.0	pres notPreg	0.0	0–0	pres notPreg
DL18-269	2019-08-03	0.1	pres notPreg	1.0	0.0	pres notPreg	0.0	0–0	pres notPreg
DL18-312	2018-11-14	5.0	pres notPreg	1.0	0.0	pres notPreg	0.1	0–0.2	pres notPreg
DL18-334	2018-11-14	0.5	pres notPreg	1.0	0.0	pres notPreg	0.0	0–0.1	pres notPreg
DL19-055	2019-07-04	0.1	pres notPreg	1.0	0.0	pres notPreg	0.0	0–0	pres notPreg
DL19-136	2019-07-05	0.6	pres notPreg	1.0	0.0	pres notPreg	0.0	0–0.1	pres notPreg
DL19-141	2019-07-02	0.4	pres notPreg	1.0	0.0	pres notPreg	0.0	0–0.1	pres notPreg
DL19-201	2019-07-05	0.4	pres notPreg	1.0	0.0	pres notPreg	0.0	0–0.1	pres notPreg
DL19-202	2019-07-05	17.4	pres notPreg	1.0	0.0	pres notPreg	0.2	0.1–0.5	pres notPreg
DL19-203	2019-07-05	633	pres preg	0.0	1.0	pres preg	0.9	0.7–1	pres preg
DL19-290	2019-11-13	0.1	pres notPreg	1.0	0.0	pres notPreg	0.0	0–0	pres notPreg
DL2011-015	2011-07-03	1.1	pres notPreg	1.0	0.0	pres notPreg	0.0	0–0.1	pres notPreg
DL2011-143	2011-11-09	436	pres preg	0.0	1.0	pres preg	0.9	0.7–1	pres preg
DL2011-152	2011-10-10	573	pres preg	0.0	1.0	pres preg	0.9	0.7–1	pres preg
DL2011-198	2011-06-12	1.2	pres notPreg	1.0	0.0	pres notPreg	0.0	0–0.1	pres notPreg
DL2011-201	2011-07-10	0.1	pres notPreg	1.0	0.0	pres notPreg	0.0	0–0	pres notPreg
DL2011-204	2011-06-18	389	pres preg	0.0	1.0	pres preg	0.8	0.6–1	pres preg
DL2011-215	2011-06-18	1079	pres preg	0.0	1.0	pres preg	0.9	0.8–1	pres preg
DL2011-216	2011-06-09	985	pres preg	0.0	1.0	pres preg	0.9	0.8–1	pres preg
DL2011-288	2011-06-17	0.6	pres notPreg	1.0	0.0	pres notPreg	0.0	0–0.1	pres notPreg
**DL2011-st-04**	2011-06-23	**74.7**	**pres notPreg**	**0.3**	**0.7**	**pres preg**	**0.6**	**0.3–0.8**	**pres preg**

DL2013-Sani-01	2013-06-07	278	pres preg	0.0	1.0	pres preg	0.8	0.6–1	pres preg
DLN01-10	2001-08-04	1.1	pres notPreg	1.0	0.0	pres notPreg	0.0	0–0.1	pres notPreg
DLN01-11	2001-08-04	0.9	pres notPreg	1.0	0.0	pres notPreg	0.0	0–0.1	pres notPreg

Table B 3 Estimated probability of being pregnant for St. Lawrence Estuary belugas biopsied between 2013 and 2016, Quebec, Canada. Three statistical approaches were compared to assign a probability of being pregnant: a 100-ng g^{-1} threshold, a model-based clustering and a logistic regression of progesterone concentrations (ng g^{-1} of tissue). For each animal, it is reported progesterone concentrations (ng g^{-1} of tissue), and reproductive status based on model classification for each statistical approach. All samples were from females of unknown reproductive status. Bold characters indicate different classifications.

a	Sampling date	Colour	% lipid	Progesterone	100 ng g^{-1} threshold	Mixture model				Logistic regression		
				(ng g^{-1} tissue)	Classification	Prob. notPreg	Prob. intermediate	Prob. pregnant	Classification	Mean Prob.	95% CI	Classification
**DLB1312**	2013-09-04	**white**	**12.7**	**15.1**	**pres notPreg**	**0.0**	**1.0**	**0.0**	**intermediate**	**0.2**	**0.1–0.4**	**pres notPreg**
DLB1314	2013-09-06	white	27.6	163	pres preg	0.0	0.1	0.9	pres preg	0.7	0.5–0.9	pres preg
DLB1315	2013-09-06	offwhite	12.9	153	pres preg	0.0	0.2	0.8	pres preg	0.7	0.5–0.9	pres preg
DLB1321	2013-09-10	offwhite	42.0	312	pres preg	0.0	0.0	1.0	pres preg	0.8	0.6–1	pres preg
DLB1322	2013-09-13	offwhite	4.9	146	pres preg	0.0	0.2	0.8	pres preg	0.7	0.5–0.9	pres preg
DLB1326	2013-09-16	white	20.3	221	pres preg	0.0	0.1	0.9	pres preg	0.8	0.5–0.9	pres preg
DLB1327	2013-09-16	white	55.8	137	pres preg	0.0	0.2	0.8	pres preg	0.7	0.4–0.9	pres preg
DLB1403	2014-09-05	white	1.8	0.5	pres notPreg	1.0	0.0	0.0	pres notPreg	0.0	0–0.1	pres notPreg
**DLB1404**	2014-09-05	**offwhite**	**3.9**	**23.8**	**pres notPreg**	**0.0**	**1.0**	**0.0**	**intermediate**	**0.3**	**0.1–0.5**	**pres notPreg**
DLB1406	2014-09-05	grey	49.2	213	pres preg	0.0	0.1	0.9	pres preg	0.8	0.5–0.9	pres preg
DLB1407	2014-09-05	white	26.1	114	pres preg	0.0	0.4	0.6	pres preg	0.6	0.4–0.9	pres preg
DLB1408	2014-09-05	white	5.1	1.0	pres notPreg	1.0	0.0	0.0	pres notPreg	0.0	0–0.1	pres notPreg
**DLB1409**	2014-09-05	**grey**	**14.9**	**25.6**	**pres notPreg**	**0.0**	**1.0**	**0.0**	**intermediate**	**0.3**	**0.1–0.5**	**pres notPreg**
DLB1410	2014-09-07	grey	18.8	0.7	pres notPreg	1.0	0.0	0.0	pres notPreg	0.0	0–0.1	pres notPreg
DLB1412	2014-09-07	white	47.1	420	pres preg	0.0	0.0	1.0	pres preg	0.9	0.6–1	pres preg
**DLB1415**	2014-09-08	**offwhite**	**25.1**	**70.8**	**pres notPreg**	**0.0**	**0.9**	**0.1**	**intermediate**	**0.5**	**0.3–0.8**	**pres preg**
DLB1416	2014-09-08	white	60.1	154	pres preg	0.0	0.2	0.8	pres preg	0.7	0.5–0.9	pres preg
**DLB1417**	2014-09-08	**white**	**3.1**	**20.0**	**pres notPreg**	**0.0**	**1.0**	**0.0**	**intermediate**	**0.3**	**0.1–0.5**	**pres notPreg**
**DLB1418**	2014-09-09	**white**	**2.9**	**20.6**	**pres notPreg**	**0.0**	**1.0**	**0.0**	**intermediate**	**0.3**	**0.1–0.5**	**pres notPreg**
**DLB1419**	2014-09-09	**white**	**6.7**	**19.0**	**pres notPreg**	**0.0**	**1.0**	**0.0**	**intermediate**	**0.3**	**0.1–0.5**	**pres notPreg**
**DLB1422**	2014-09-10	**white**	**5.3**	**3.9**	**pres notPreg**	**0.4**	**0.6**	**0.0**	**intermediate**	**0.1**	**0–0.2**	**pres notPreg**
**DLB1423**	2014-09-10	**offwhite**	**4.9**	**60.9**	**pres notPreg**	**0.0**	**0.9**	**0.1**	**intermediate**	**0.5**	**0.3–0.8**	**pres preg**
**DLB1424**	2014-09-10	**white**	**1.9**	**25.6**	**pres notPreg**	**0.0**	**1.0**	**0.0**	**intermediate**	**0.3**	**0.1–0.5**	**pres notPreg**
DLB1425	2014-09-10	white	13.9	0.5	pres notPreg	1.0	0.0	0.0	pres notPreg	0.0	0–0.1	pres notPreg
DLB1427	2014-09-10	grey	22.6	243	pres preg	0.0	0.0	1.0	pres preg	0.8	0.5–0.9	pres preg
DLB1428	2014-09-10	offwhite	53.2	267	pres preg	0.0	0.0	1.0	pres preg	0.8	0.6–0.9	pres preg

DLB1429	2014-09-13	grey	34.8	1.4	pres notPreg	1.0	0.0	0.0	pres notPreg	0.0	0–0.1	pres notPreg
**DLB1430**	2014-09-13	**white**	**44.6**	**53.1**	**pres notPreg**	**0.0**	**0.9**	**0.1**	**intermediate**	**0.5**	**0.2–0.7**	**pres notPreg**
DLB1440	2014-09-15	offwhite	19.2	1.2	pres notPreg	1.0	0.0	0.0	pres notPreg	0.0	0–0.1	pres notPreg
DLB1441	2014-09-15	white	58.0	378	pres preg	0.0	0.0	1.0	pres preg	0.8	0.6–1	pres preg
DLB1443	2014-09-15	white	3.6	1.1	pres notPreg	1.0	0.0	0.0	pres notPreg	0.0	0–0.1	pres notPreg
DLB1452	2014-09-17	white	1.3	0.5	pres notPreg	1.0	0.0	0.0	pres notPreg	0.0	0–0.1	pres notPreg
DLB1520	2015-09-11	white	5.2	0.5	pres notPreg	1.0	0.0	0.0	pres notPreg	0.0	0–0.1	pres notPreg
DLB1522	2015-09-12	white	5.5	0.9	pres notPreg	1.0	0.0	0.0	pres notPreg	0.0	0–0.1	pres notPreg
DLB1524	2015-09-12	white	1.7	0.7	pres notPreg	1.0	0.0	0.0	pres notPreg	0.0	0–0.1	pres notPreg
**DLB1526**	2015-09-12	**grey**	**2.3**	**23.5**	**pres notPreg**	**0.0**	**1.0**	**0.0**	**intermediate**	**0.3**	**0.1–0.5**	**pres notPreg**
**DLB1527**	2015-09-12	**white**	**7.9**	**65.6**	**pres notPreg**	**0.0**	**0.9**	**0.1**	**intermediate**	**0.5**	**0.3–0.8**	**pres preg**
DLB1529	2015-09-17	grey	4.1	1.2	pres notPreg	1.0	0.0	0.0	pres notPreg	0.0	0–0.1	pres notPreg
**DLB1530**	2015-09-18	**offwhite**	**9.6**	**60.2**	**pres notPreg**	**0.0**	**0.9**	**0.1**	**intermediate**	**0.5**	**0.3–0.7**	**pres preg**
DLB1555	2015-09-24	grey	51.6	0.3	pres notPreg	1.0	0.0	0.0	pres notPreg	0.0	0–0	pres notPreg
DLB1558	2015-09-24	white	63.8	305	pres preg	0.0	0.0	1.0	pres preg	0.8	0.6–1	pres preg
DLB1610	2016-09-09	offwhite	35.5	1.6	pres notPreg	1.0	0.0	0.0	pres notPreg	0.0	0–0.1	pres notPreg
DLB1612	2016-09-09	white	30.7	417	pres preg	0.0	0.0	1.0	pres preg	0.8	0.6–1	pres preg
DLB1614	2016-09-10	grey	27.3	306	pres preg	0.0	0.0	1.0	pres preg	0.8	0.6–1	pres preg
**DLB1617**	2016-09-12	**grey**	**26.4**	**46.6**	**pres notPreg**	**0.0**	**1.0**	**0.0**	**intermediate**	**0.4**	**0.2–0.7**	**pres notPreg**
DLB1618	2016-09-12	offwhite	39.3	464	pres preg	0.0	0.0	1.0	pres preg	0.9	0.6–1	pres preg
DLB1620	2016-09-13	white	41.3	268	pres preg	0.0	0.0	1.0	pres preg	0.8	0.6–0.9	pres preg
DLB1621	2016-09-13	white	2.3	1.4	pres notPreg	1.0	0.0	0.0	pres notPreg	0.0	0–0.1	pres notPreg
**DLB1622**	2016-09-15	**white**	**36.0**	**9.3**	**pres notPreg**	**0.0**	**1.0**	**0.0**	**intermediate**	**0.2**	**0–0.3**	**pres notPreg**
DLB1624	2016-09-15	white	43.3	210	pres preg	0.0	0.1	0.9	pres preg	0.8	0.5–0.9	pres preg
DLB1628	2016-09-17	offwhite	30.7	0.9	pres notPreg	1.0	0.0	0.0	pres notPreg	0.0	0–0.1	pres notPreg
DLB1631	2016-09-19	white	31.5	2.3	pres notPreg	0.9	0.1	0.0	pres notPreg	0.1	0–0.1	pres notPreg
**DLB1632**	2016-09-19	**grey**	**1.1**	**14.2**	**pres notPreg**	**0.0**	**1.0**	**0.0**	**intermediate**	**0.2**	**0.1–0.4**	**pres notPreg**
DLB1633	2016-09-19	grey	39.6	238	pres preg	0.0	0.1	0.9	pres preg	0.8	0.5–0.9	pres preg
**DLB1634**	2016-09-19	**grey**	**31.7**	**8.6**	**pres notPreg**	**0.0**	**1.0**	**0.0**	**intermediate**	**0.1**	**0–0.3**	**pres notPreg**
**DLB1635**	2016-09-20	**white**	**58.1**	**52.7**	**pres notPreg**	**0.0**	**0.9**	**0.1**	**intermediate**	**0.5**	**0.2–0.7**	**pres notPreg**
DLB1639	2016-09-21	white	19.0	185	pres preg	0.0	0.1	0.9	pres preg	0.7	0.5–0.9	pres preg
DLB1641	2016-09-21	grey	44.5	0.6	pres notPreg	1.0	0.0	0.0	pres notPreg	0.0	0–0.1	pres notPreg
DLB1642	2016-09-22	white	10.5	0.6	pres notPreg	1.0	0.0	0.0	pres notPreg	0.0	0–0.1	pres notPreg
**DLB1646**	2016-09-22	**white**	**24.4**	**17.1**	**pres notPreg**	**0.0**	**1.0**	**0.0**	**intermediate**	**0.2**	**0.1–0.4**	**pres notPreg**
**DLB1647**	2016-09-22	**white**	**47.2**	**30.4**	**pres notPreg**	**0.0**	**1.0**	**0.0**	**intermediate**	**0.3**	**0.1–0.6**	**pres notPreg**

DLB1648	2016-09-22	white	50.9	246	pres preg	0.0	0.0	1.0	pres preg	0.8	0.5–0.9	pres preg
DLB1649	2016-09-22	grey	26.7	0.6	pres notPreg	1.0	0.0	0.0	pres notPreg	0.0	0–0.1	pres notPreg
**DLB1650**	2016-09-22	**white**	**6.2**	**67.4**	**pres notPreg**	**0.0**	**0.9**	**0.1**	**intermediate**	**0.5**	**0.3–0.8**	**pres preg**
**DLB1652**	2016-09-22	**white**	**21.4**	**39.6**	**pres notPreg**	**0.0**	**1.0**	**0.0**	**intermediate**	**0.4**	**0.2–0.6**	**pres notPreg**

Table B 4 Estimated probability of being pregnant for St. Lawrence Estuary belugas biopsied between 2013 and 2016, Quebec, Canada. Similar to Table B 3, three statistical approaches were compared to assign a probability of being pregnant, but samples with low lipid content (<5%) were excluded from analyses (N=51). For each animal, it is reported progesterone concentrations (ng g^{-1} of tissue), and reproductive status based on model classification for each statistical approach. All samples were from females of unknown reproductive status. Bold characters indicate different classifications.

Sample	Sampling date	Colour	% Lipid	Progesterone	100 ng g^{-1} threshold	Mixture model				Logistic regression		
				(ng g^{-1} tissue)	Classification	Prob. notPreg	Prob. intermediate	Prob. pregnant	Classification	Mean Prob.	95% CI	Classification
**DLB1312**	2013-09-04	**White**	**12.7**	**15.1**	**pres notPreg**	**0.0**	**1.0**	**0.0**	**intermediate**	**0.2**	**0.1–0.4**	**pres notPreg**
DLB1314	2013-09-06	White	27.6	163	pres preg	0.0	0.2	0.8	pres preg	0.7	0.5–0.9	pres preg
DLB1315	2013-09-06	offwhite	12.9	153	pres preg	0.0	0.3	0.7	pres preg	0.7	0.5–0.9	pres preg
DLB1321	2013-09-10	offwhite	42.0	312	pres preg	0.0	0.1	0.9	pres preg	0.8	0.6–1	pres preg
DLB1326	2013-09-16	White	20.3	221	pres preg	0.0	0.1	0.9	pres preg	0.8	0.5–0.9	pres preg
DLB1327	2013-09-16	White	55.8	137	pres preg	0.0	0.4	0.6	pres preg	0.7	0.4–0.9	pres preg
DLB1406	2014-09-05	Grey	49.2	213	pres preg	0.0	0.1	0.9	pres preg	0.8	0.5–0.9	pres preg
**DLB1407**	2014-09-05	**White**	**26.1**	**114**	**pres preg**	**0.0**	**0.6**	**0.4**	**intermediate**	**0.6**	**0.4–0.9**	**pres preg**
DLB1408	2014-09-05	White	5.1	1.0	pres notPreg	1.0	0.0	0.0	pres notPreg	0.0	0–0.1	pres notPreg
**DLB1409**	2014-09-05	**Grey**	**14.9**	**25.6**	**pres notPreg**	**0.0**	**1.0**	**0.0**	**intermediate**	**0.3**	**0.1–0.5**	**pres notPreg**
DLB1410	2014-09-07	Grey	18.8	0.7	pres notPreg	1.0	0.0	0.0	pres notPreg	0.0	0–0.1	pres notPreg
DLB1412	2014-09-07	White	47.1	420	pres preg	0.0	0.1	0.9	pres preg	0.9	0.7–1	pres preg
**DLB1415**	2014-09-08	**offwhite**	**25.1**	**70.8**	**pres notPreg**	**0.0**	**0.9**	**0.1**	**intermediate**	**0.5**	**0.3–0.8**	**pres preg**
DLB1416	2014-09-08	White	60.1	154	pres preg	0.0	0.3	0.7	pres preg	0.7	0.5–0.9	pres preg
**DLB1419**	2014-09-09	**White**	**6.7**	**19.0**	**pres notPreg**	**0.0**	**1.0**	**0.0**	**intermediate**	**0.2**	**0.1–0.4**	**pres notPreg**
DLB1422	2014-09-10	White	5.3	3.9	pres notPreg	0.5	0.5	0.0	pres notPreg	0.1	0–0.2	pres notPreg
DLB1425	2014-09-10	White	13.9	0.5	pres notPreg	1.0	0.0	0.0	pres notPreg	0.0	0–0	pres notPreg
DLB1427	2014-09-10	Grey	22.6	243	pres preg	0.0	0.1	0.9	pres preg	0.8	0.6–0.9	pres preg
DLB1428	2014-09-10	offwhite	53.2	267	pres preg	0.0	0.1	0.9	pres preg	0.8	0.6–0.9	pres preg
DLB1429	2014-09-13	Grey	34.8	1.4	pres notPreg	1.0	0.0	0.0	pres notPreg	0.0	0–0.1	pres notPreg
**DLB1430**	2014-09-13	**White**	**44.6**	**53.1**	**pres notPreg**	**0.0**	**1.0**	**0.0**	**intermediate**	**0.5**	**0.2–0.7**	**pres notPreg**
DLB1440	2014-09-15	offwhite	19.2	1.2	pres notPreg	1.0	0.0	0.0	pres notPreg	0.0	0–0.1	pres notPreg
DLB1441	2014-09-15	White	58.0	378	pres preg	0.0	0.1	0.9	pres preg	0.8	0.6–1	pres preg
DLB1520	2015-09-11	White	5.2	0.5	pres notPreg	1.0	0.0	0.0	pres notPreg	0.0	0–0.1	pres notPreg
DLB1522	2015-09-12	White	5.5	0.9	pres notPreg	1.0	0.0	0.0	pres notPreg	0.0	0–0.1	pres notPreg

**DLB1527**	2015-09-12	**White**	**7.9**	**65.6**	**pres notPreg**	**0.0**	**0.9**	**0.1**	**intermediate**	**0.5**	**0.3–0.8**	**pres preg**
**DLB1530**	2015-09-18	**offwhite**	**9.6**	**60.2**	**pres notPreg**	**0.0**	**0.9**	**0.1**	**intermediate**	**0.5**	**0.3–0.7**	**pres notPreg**
DLB1555	2015-09-24	Grey	51.6	0.3	pres notPreg	1.0	0.0	0.0	pres notPreg	0.0	0–0	pres notPreg
DLB1558	2015-09-24	White	63.7	305	pres preg	0.0	0.1	0.9	pres preg	0.8	0.6–1	pres preg
DLB1610	2016-09-09	offwhite	35.5	1.6	pres notPreg	0.9	0.1	0.0	pres notPreg	0.0	0–0.1	pres notPreg
DLB1612	2016-09-09	White	30.7	417	pres preg	0.0	0.1	0.9	pres preg	0.9	0.7–1	pres preg
DLB1614	2016-09-10	Grey	27.3	306	pres preg	0.0	0.1	0.9	pres preg	0.8	0.6–1	pres preg
**DLB1617**	2016-09-12	**Grey**	**26.4**	**46.6**	**pres notPreg**	**0.0**	**1.0**	**0.0**	**intermediate**	**0.4**	**0.2–0.7**	**pres notPreg**
DLB1618	2016-09-12	offwhite	39.3	465	pres preg	0.0	0.1	0.9	pres preg	0.9	0.7–1	pres preg
DLB1620	2016-09-13	White	41.3	268	pres preg	0.0	0.1	0.9	pres preg	0.8	0.6–0.9	pres preg
**DLB1622**	2016-09-15	**White**	**36.0**	**9.3**	**pres notPreg**	**0.1**	**0.9**	**0.0**	**intermediate**	**0.1**	**0–0.3**	**pres notPreg**
DLB1624	2016-09-15	White	43.3	210	pres preg	0.0	0.1	0.9	pres preg	0.8	0.5–0.9	pres preg
DLB1628	2016-09-17	offwhite	30.7	0.9	pres notPreg	1.0	0.0	0.0	pres notPreg	0.0	0–0.1	pres notPreg
DLB1631	2016-09-19	White	31.5	2.3	pres notPreg	0.8	0.2	0.0	pres notPreg	0.0	0–0.1	pres notPreg
DLB1633	2016-09-19	Grey	39.6	238	pres preg	0.0	0.1	0.9	pres preg	0.8	0.6–0.9	pres preg
**DLB1634**	2016-09-19	**Grey**	**31.7**	**8.6**	**pres notPreg**	**0.1**	**0.9**	**0.0**	**intermediate**	**0.1**	**0–0.3**	**pres notPreg**
**DLB1635**	2016-09-20	**White**	**58.1**	**52.7**	**pres notPreg**	**0.0**	**1.0**	**0.0**	**intermediate**	**0.5**	**0.2–0.7**	**pres notPreg**
DLB1639	2016-09-21	White	18.9	185	pres preg	0.0	0.2	0.8	pres preg	0.7	0.5–0.9	pres preg
DLB1641	2016-09-21	Grey	44.5	0.6	pres notPreg	1.0	0.0	0.0	pres notPreg	0.0	0–0.1	pres notPreg
DLB1642	2016-09-22	White	10.5	0.6	pres notPreg	1.0	0.0	0.0	pres notPreg	0.0	0–0.1	pres notPreg
**DLB1646**	2016-09-22	**White**	**24.4**	**17.1**	**pres notPreg**	**0.0**	**1.0**	**0.0**	**intermediate**	**0.2**	**0.1–0.4**	**pres notPreg**
**DLB1647**	2016-09-22	**White**	**47.2**	**30.4**	**pres notPreg**	**0.0**	**1.0**	**0.0**	**intermediate**	**0.3**	**0.1–0.6**	**pres notPreg**
DLB1648	2016-09-22	White	50.9	246	pres preg	0.0	0.1	0.9	pres preg	0.8	0.6–0.9	pres preg
DLB1649	2016-09-22	Grey	26.7	0.6	pres notPreg	1.0	0.0	0.0	pres notPreg	0.0	0–0.1	pres notPreg
**DLB1650**	2016-09-22	**White**	**6.2**	**67.4**	**pres notPreg**	**0.0**	**0.9**	**0.1**	**intermediate**	**0.5**	**0.3–0.8**	**pres preg**
**DLB1652**	2016-09-22	**White**	**21.4**	**39.6**	**pres notPreg**	**0.0**	**1.0**	**0.0**	**intermediate**	**0.4**	**0.2–0.6**	**pres notPreg**

Appendix B. Supplementary results, part II: Individual probabilities of pregnancy as estimated by three approaches, based on blubber progesterone expressed in ng g^{-1} of lipid.

Table B 5 Estimated probability of being pregnant for St. Lawrence Estuary beluga necropsied between 1997 and 2019, Quebec, Canada, based on blubber progesterone concentration corrected for sample lipid content. Three statistical approaches were compared to assign a probability of being pregnant: a 150-ng g^{-1} threshold, a model-based clustering and a logistic regression of progesterone concentrations (ng g^{-1} of lipid). For each animal, it is reported sex, age, progesterone concentrations (ng g^{-1} of lipid), known reproductive status based on examination of reproductive tracts, sexual maturity (mature, immature), and reproductive status based on model classification for each statistical approach. Bold characters indicate inaccurate classification.

Sample	Sampling date	Sex	Age	Progesterone	Reproductive status	Maturity	150 ng g^{-1} threshold	Mixture model			Logistic regression		
				(ng g^{-1} lipid)			Classification	Prob. notPreg	Prob. pregnant	Classification	Mean Prob.	95% CI	Classification
DL1997-002	1997-05-23	F	57	1.7	lactating	M	pres notPreg	1.0	0.0	pres notPreg	0.0	0-0.1	pres notPreg
DL1997-003	1997-06-08	F	43	715	pregnant	M	pres preg	0.0	1.0	pres preg	0.9	0.7-1	pres preg
DL1997-005	1997-07-15	M	12	2.5	-	I	pres notPreg	1.0	0.0	pres notPreg	0.0	0-0.1	pres notPreg
DL1997-006	1997-07-29	F	62	0.4	resting	M	pres notPreg	1.0	0.0	pres notPreg	0.0	0-0	pres notPreg
DL1998-003	1998-05-24	F	59	17.9	resting	M	pres notPreg	1.0	0.0	pres notPreg	0.2	0-0.4	pres notPreg
DL1998-004	1998-05-24	F	42	1.9	resting	M	pres notPreg	1.0	0.0	pres notPreg	0.0	0-0.1	pres notPreg
DL1998-007	1998-08-07	M	36	1.4	-	M	pres notPreg	1.0	0.0	pres notPreg	0.0	0-0.1	pres notPreg
DL1999-009	1999-10-15	F	39	3.3	resting	M	pres notPreg	1.0	0.0	pres notPreg	0.0	0-0.1	pres notPreg
DL2000-004	2000-10-06	F	-	5.1	lactating	M	pres notPreg	1.0	0.0	pres notPreg	0.1	0-0.2	pres notPreg
DL2000-005	2000-10-12	F	50	15.6	lactating	M	pres notPreg	1.0	0.0	pres notPreg	0.2	0-0.3	pres notPreg
DL2001-005	2001-09-25	M	7	5.2	-	I	pres notPreg	1.0	0.0	pres notPreg	0.1	0-0.2	pres notPreg
DL2002-005	2002-07-25	F	27	308	pregnant	M	pres preg	0.0	1.0	pres preg	0.7	0.5-0.9	pres preg
DL2003-002	2003-06-13	F	45	13.5	pregnant	M	**pres notPreg**	**1.0**	**0.0**	**pres notPreg**	**0.1**	**0-0.3**	**pres notPreg**
DL2003-006	2003-10-17	F	52	5.1	resting	M	pres notPreg	1.0	0.0	pres notPreg	0.1	0-0.2	pres notPreg
DL2003-007	2003-10-22	F	30	0.2	lactating	M	pres notPreg	1.0	0.0	pres notPreg	0.0	0-0	pres notPreg
DL2004-003	2004-06-11	F	56	1.2	lactating	M	pres notPreg	1.0	0.0	pres notPreg	0.0	0-0.1	pres notPreg
DL2004-004	2004-06-19	M	3	0.3	-	I	pres notPreg	1.0	0.0	pres notPreg	0.0	0-0	pres notPreg
DL2005-004	2005-09-04	M	50	0.3	-	M	pres notPreg	1.0	0.0	pres notPreg	0.0	0-0	pres notPreg
DL2006-002	2006-09-17	F	4	0.5	-	I	pres notPreg	1.0	0.0	pres notPreg	0.0	0-0	pres notPreg
DL2007-002	2007-05-27	F	44	1.4	lactating	M	pres notPreg	1.0	0.0	pres notPreg	0.0	0-0.1	pres notPreg
DL2007-004	2007-07-03	F	28	316	recently gave birth	M	pres preg	0.0	1.0	pres preg	0.8	0.5-0.9	pres preg
DL2007-008	2007-09-11	F	38	0.5	lactating	M	pres notPreg	1.0	0.0	pres notPreg	0.0	0-0	pres notPreg
DL2007-009	2007-09-22	M	50	2.7	-	M	pres notPreg	1.0	0.0	pres notPreg	0.0	0-0.1	pres notPreg

DL2008-003	2008-06-16	F	52	8.2	lactating	M	pres notPreg	1.0	0.0	pres notPreg	0.1	0-0.2	pres notPreg
DL2008-007	2008-09-18	F	57	1.7	lactating	M	pres notPreg	1.0	0.0	pres notPreg	0.0	0-0.1	pres notPreg
DL2009-001	2009-06-16	F	12	618	lactating	M	**pres preg**	**0.0**	**1.0**	**pres preg**	**0.8**	**0.6-1**	**pres preg**
DL2010-001	2010-03-09	F	2	0.4	-	I	pres notPreg	1.0	0.0	pres notPreg	0.0	0-0	pres notPreg
DL2010-002	2010-03-21	F	1	0.7	-	I	pres notPreg	1.0	0.0	pres notPreg	0.0	0-0.1	pres notPreg
DL2010-005	2010-06-10	F	51	460	pregnant	M	pres preg	0.0	1.0	pres preg	0.8	0.6-1	pres preg
DL2010-006	2010-07-16	F	18	268	recently gave birth	M	pres preg	0.0	1.0	pres preg	0.7	0.5-0.9	pres preg
DL2010-007	2010-07-31	F	40	573	pregnant	M	pres preg	0.0	1.0	pres preg	0.8	0.6-1	pres preg
DL2010-008	2010-08-29	F	31	283	pregnant	M	pres preg	0.0	1.0	pres preg	0.7	0.5-0.9	pres preg
DL2010-011	2010-12-31	F	35	3.3	resting	M	pres notPreg	1.0	0.0	pres notPreg	0.1	0-0.1	pres notPreg
DL2011-001	2011-04-07	M	2	1.2	-	I	pres notPreg	1.0	0.0	pres notPreg	0.0	0-0.1	pres notPreg
DL2011-002	2011-05-19	F	44	1257	pregnant	M	pres preg	0.0	1.0	pres preg	0.9	0.7-1	pres preg
DL2011-003	2011-06-08	F	30	1315	pregnant	M	pres preg	0.0	1.0	pres preg	0.9	0.7-1	pres preg
DL2011-004	2011-07-12	F	56	25.6	lactating	M	pres notPreg	0.9	0.1	pres notPreg	0.2	0.1-0.4	pres notPreg
DL2011-005	2011-08-09	F	14	406	pregnant	M	pres preg	0.0	1.0	pres preg	0.8	0.6-0.9	pres preg
DL2011-006	2011-09-10	F	68	0.9	resting	M	pres notPreg	1.0	0.0	pres notPreg	0.0	0-0.1	pres notPreg
DL2011-008	2011-10-28	F	22	129	lactating	M	pres notPreg	**0.0**	**1.0**	**pres preg**	**0.6**	**0.3-0.8**	**pres preg**
DL2011-009	2011-11-09	M	43	0.2	-	M	pres notPreg	1.0	0.0	pres notPreg	0.0	0-0	pres notPreg
DL2012-001	2012-05-19	F	26	110	recently gave birth	M	**pres notPreg**	0.1	0.9	pres preg	0.5	0.3-0.8	pres preg
DL2012-003	2012-07-17	F	21	658	recently gave birth	M	pres preg	0.0	1.0	pres preg	0.8	0.6-1	pres preg
DL2013-005	2013-09-15	M	21	1.2	-	M	pres notPreg	1.0	0.0	pres notPreg	0.0	0-0.1	pres notPreg
DL2014-003	2014-09-28	F	32	82.5	lactating	M	pres notPreg	**0.2**	**0.8**	**pres preg**	0.5	0.2-0.7	pres notPreg
DL2015-002	2015-05-31	M	6	0.1	-	I	pres notPreg	1.0	0.0	pres notPreg	0.0	0-0	pres notPreg
DL2015-003	2015-07-17	F	21	452	pregnant	M	pres preg	0.0	1.0	pres preg	0.8	0.6-1	pres preg
DL2015-006	2015-08-18	F	33	245	recently gave birth	M	pres preg	0.0	1.0	pres preg	0.7	0.5-0.9	pres preg
DL2017-001	2017-05-20	F	53	9.4	lactating	M	pres notPreg	1.0	0.0	pres notPreg	0.1	0-0.3	pres notPreg
DL2017-003	2017-09-20	F	43	180	recently gave birth	M	pres preg	0.0	1.0	pres preg	0.6	0.4-0.9	pres preg
DL2017-004	2017-10-23	F	45	71.0	lactating	M	pres notPreg	**0.3**	**0.7**	**pres preg**	0.4	0.2-0.7	pres notPreg
DL2018-001	2018-05-20	F	47	1.8	lactating	M	pres notPreg	1.0	0.0	pres notPreg	0.0	0-0.1	pres notPreg
DL2018-002	2018-07-15	F	18	385	recently gave birth	M	pres preg	0.0	1.0	pres preg	0.8	0.6-0.9	pres preg
DL2018-003	2018-07-22	F	36	873	recently gave birth	M	pres preg	0.0	1.0	pres preg	0.9	0.7-1	pres preg
DL2018-004	2018-07-29	F	45	3.9	lactating	M	pres notPreg	1.0	0.0	pres notPreg	0.1	0-0.2	pres notPreg
DL2019-001	2019-04-08	F	5	2.0	-	I	pres notPreg	1.0	0.0	pres notPreg	0.0	0-0.1	pres notPreg
DL2019-002	2019-05-13	M	2	0.2	-	I	pres notPreg	1.0	0.0	pres notPreg	0.0	0-0	pres notPreg
DL2019-004	2019-07-04	F	33	467	pregnant	M	pres preg	0.0	1.0	pres preg	0.8	0.6-1	pres preg

DL2019-005	2019-07-16	F	22	405	pregnant	M	pres preg	0.0	1.0	pres preg	0.8	0.6-0.9	pres preg
DL2019-006	2019-08-01	F	48	7.2	**recently gave birth**	**M**	**pres notPreg**	**1.0**	**0.0**	**pres notPreg**	**0.1**	**0-0.2**	**pres notPreg**
DL2019-008	2019-10-17	F	30	1041	recently gave birth	M	pres preg	0.0	1.0	pres preg	0.9	0.7-1	pres preg
DL2019-009	2019-11-09	F	28	454	pregnant	M	pres preg	0.0	1.0	pres preg	0.8	0.6-1	pres preg

Table B 6 Estimated probability of being pregnant for Nunavik beluga whales hunted between 2001 and 2019, Quebec, Canada, based on model-based clustering of progesterone concentrations corrected for sample lipid content. Three statistical approaches were compared to assign a probability of being pregnant: a 150 ng g^{-1} threshold, a model-based clustering and a logistic regression of progesterone concentrations (ng g^{-1} of lipid). For each animal, it is reported progesterone concentrations (ng g^{-1} of lipid), probability of a whale to be non-pregnant or pregnant, and reproductive status based on model classification for each statistical approach. All samples were from females of unknown reproductive status. Bold characters indicate different classifications.

**Sample**	**Sampling date**	**Progesterone**	**150 ng g^{-1}**	**Cluster-based**			**Logistic regression**		
		**(ng g^{-1} lipid)**	**Classification**	**Prob. Not**	**Prob.**	**Classification**	**Mean**	**95% CI**	**Classification**
DL-10001	2008-06-09	798	pres preg	0.0	1.0	pres preg	0.9	0.7-1	pres preg
DL-10002	2008-06-11	747	pres preg	0.0	1.0	pres preg	0.9	0.7-1	pres preg
DL-10004	2008-06-15	1.3	pres notPreg	1.0	0.0	pres notPreg	0.0	0-0.1	pres notPreg
DL-10008	2008-06-10	1257	pres preg	0.0	1.0	pres preg	0.9	0.8-1	pres preg
DL-10012	2008-06-10	276	pres preg	0.0	1.0	pres preg	0.7	0.5-0.9	pres preg
DL-10014	2008-06-15	0.4	pres notPreg	1.0	0.0	pres notPreg	0.0	0-0	pres notPreg
DL-10102	2008-06-19	2.2	pres notPreg	1.0	0.0	pres notPreg	0.0	0-0.1	pres notPreg
DL-10103	2008-06-23	1433	pres preg	0.0	1.0	pres preg	0.9	0.8-1	pres preg
DL-10105	2008-06-19	287	pres preg	0.0	1.0	pres preg	0.7	0.5-0.9	pres preg
DL-8047	2016-11-10	0.6	pres notPreg	1.0	0.0	pres notPreg	0.0	0-0	pres notPreg
DL-9076	2007-09-19	2.9	pres notPreg	1.0	0.0	pres notPreg	0.0	0-0.1	pres notPreg
DL-9129	2007-06-29	3.7	pres notPreg	1.0	0.0	pres notPreg	0.1	0-0.1	pres notPreg
DL-9140	2007-06-27	3.1	pres notPreg	1.0	0.0	pres notPreg	0.0	0-0.1	pres notPreg
DL-9143	2007-06-24	1192	pres preg	0.0	1.0	pres preg	0.9	0.7-1	pres preg
DL-9156	2007-06-22	12.4	pres notPreg	1.0	0.0	pres notPreg	0.1	0-0.3	pres notPreg
DL-9164	2010-06-15	352	pres preg	0.0	1.0	pres preg	0.8	0.6-0.9	pres preg
DL-9206	2009-10-11	349	pres preg	0.0	1.0	pres preg	0.8	0.6-0.9	pres preg
DL-9215	2007-07-21	232	pres preg	0.0	1.0	pres preg	0.7	0.5-0.9	pres preg
DL-9217	2007-07-22	0.2	pres notPreg	1.0	0.0	pres notPreg	0.0	0-0	pres notPreg
DL09-174	2009-07-25	288	pres preg	0.0	1.0	pres preg	0.7	0.5-0.9	pres preg
DL09-183	2009-07-07	279	pres preg	0.0	1.0	pres preg	0.7	0.5-0.9	pres preg
DL09-212	2009-06-26	0.2	pres notPreg	1.0	0.0	pres notPreg	0.0	0-0	pres notPreg
DL09-27	2009-10-17	834	pres preg	0.0	1.0	pres preg	0.9	0.7-1	pres preg
DL09-71	2009-06-21	0.7	pres notPreg	1.0	0.0	pres notPreg	0.0	0-0.1	pres notPreg
DL09-74	2009-06-20	0.5	pres notPreg	1.0	0.0	pres notPreg	0.0	0-0	pres notPreg
DL09-81	-	80.8	pres notPreg	0.6	0.4	pres notPreg	0.5	0.2-0.7	pres notPreg
DL09-86	2009-06-24	4.4	pres notPreg	1.0	0.0	pres notPreg	0.1	0-0.2	pres notPreg
DL09-88	2009-06-27	1.8	pres notPreg	1.0	0.0	pres notPreg	0.0	0-0.1	pres notPreg

DL10-134	2010-10-12	1.6	pres notPreg	1.0	0.0	pres notPreg	0.0	0-0.1	pres notPreg
DL10-169	2010-11-06	783	pres preg	0.0	1.0	pres preg	0.9	0.7-1	pres preg
DL10-173	2010-06-29	755	pres preg	0.0	1.0	pres preg	0.9	0.7-1	pres preg
DL10-202	2010-06-17	8.4	pres notPreg	1.0	0.0	pres notPreg	0.1	0-0.2	pres notPreg
DL10-24	2012-06-17	698	pres preg	0.0	1.0	pres preg	0.9	0.7-1	pres preg
DL10-242	2012-07-01	2674	pres preg	0.0	1.0	pres preg	1.0	0.8-1	pres preg
DL10-8	2010-06-12	947	pres preg	0.0	1.0	pres preg	0.9	0.7-1	pres preg
DL12-053	2012-06-17	2.4	pres notPreg	1.0	0.0	pres notPreg	0.0	0-0.1	pres notPreg
DL12-154	2012-06-11	1380	pres preg	0.0	1.0	pres preg	0.9	0.8-1	pres preg
DL12-157	2012-06-12	0.2	pres notPreg	1.0	0.0	pres notPreg	0.0	0-0	pres notPreg
DL12-169	2012-06-11	701	pres preg	0.0	1.0	pres preg	0.9	0.7-1	pres preg
DL12-273	2012-06-05	991	pres preg	0.0	1.0	pres preg	0.9	0.7-1	pres preg
DL12-292	2012-10-23	1026	pres preg	0.0	1.0	pres preg	0.9	0.7-1	pres preg
DL12-293	2012-10-22	513	pres preg	0.0	1.0	pres preg	0.8	0.6-1	pres preg
DL13-020	2018-07-20	3.3	pres notPreg	1.0	0.0	pres notPreg	0.0	0-0.1	pres notPreg
DL13-026	2015-06-22	0.7	pres notPreg	1.0	0.0	pres notPreg	0.0	0-0.1	pres notPreg
DL13-055	2013-06-23	972	pres preg	0.0	1.0	pres preg	0.9	0.7-1	pres preg
DL13-085	2013-06-22	928	pres preg	0.0	1.0	pres preg	0.9	0.7-1	pres preg
DL13-096	2014-06-23	0.2	pres notPreg	1.0	0.0	pres notPreg	0.0	0-0	pres notPreg
DL13-097	2014-06-23	0.2	pres notPreg	1.0	0.0	pres notPreg	0.0	0-0	pres notPreg
DL13-099	2014-06-23	16.1	pres notPreg	1.0	0.0	pres notPreg	0.2	0-0.3	pres notPreg
DL13-109	2014-06-23	312	pres preg	0.0	1.0	pres preg	0.8	0.5-0.9	pres preg
DL13-114	2014-06-26	42.6	pres notPreg	1.0	0.0	pres notPreg	0.3	0.1-0.6	pres notPreg
DL13-116	2015-11-04	359	pres preg	0.0	1.0	pres preg	0.8	0.6-0.9	pres preg
DL13-117	2015-11-04	766	pres preg	0.0	1.0	pres preg	0.9	0.7-1	pres preg
DL13-118	2015-11-04	0.2	pres notPreg	1.0	0.0	pres notPreg	0.0	0-0	pres notPreg
DL13-125	2014-10-29	0.2	pres notPreg	1.0	0.0	pres notPreg	0.0	0-0	pres notPreg
DL13-130	2014-10-29	3.1	pres notPreg	1.0	0.0	pres notPreg	0.0	0-0.1	pres notPreg
DL13-132	2013-07-24	0.5	pres notPreg	1.0	0.0	pres notPreg	0.0	0-0	pres notPreg
DL13-182	2013-06-28	613	pres preg	0.0	1.0	pres preg	0.9	0.7-1	pres preg
DL13-185	2013-06-28	0.9	pres notPreg	1.0	0.0	pres notPreg	0.0	0-0.1	pres notPreg
DL13-191	2013-06-28	1.0	pres notPreg	1.0	0.0	pres notPreg	0.0	0-0.1	pres notPreg
DL13-192	2013-06-28	0.4	pres notPreg	1.0	0.0	pres notPreg	0.0	0-0	pres notPreg
DL13-241	2019-10-25	738	pres preg	0.0	1.0	pres preg	0.9	0.7-1	pres preg
DL13-sani-05	2013-06-07	2.3	pres notPreg	1.0	0.0	pres notPreg	0.0	0-0.1	pres notPreg
DL14-082	2014-06-17	687	pres preg	0.0	1.0	pres preg	0.9	0.7-1	pres preg

DL14-179	2014-06-12	2.8	pres notPreg	1.0	0.0	pres notPreg	0.0	0-0.1	pres notPreg
DL14-302	2014-08-18	0.8	pres notPreg	1.0	0.0	pres notPreg	0.0	0-0.1	pres notPreg
DL15-029	2015-06-27	0.4	pres notPreg	1.0	0.0	pres notPreg	0.0	0-0	pres notPreg
DL15-037	2015-06-23	241	pres preg	0.0	1.0	pres preg	0.7	0.5-0.9	pres preg
DL15-042	2015-06-23	584	pres preg	0.0	1.0	pres preg	0.8	0.6-1	pres preg
DL15-113	2015-10-28	532	pres preg	0.0	1.0	pres preg	0.8	0.6-1	pres preg
DL15-120	2015-06-26	1.1	pres notPreg	1.0	0.0	pres notPreg	0.0	0-0.1	pres notPreg
DL15-129	2015-07-07	0.5	pres notPreg	1.0	0.0	pres notPreg	0.0	0-0	pres notPreg
DL15-159	2015-10-28	9.6	pres notPreg	1.0	0.0	pres notPreg	0.1	0-0.3	pres notPreg
DL15-185	2017-06-17	1324	pres preg	0.0	1.0	pres preg	0.9	0.8-1	pres preg
DL15-187	2017-06-17	0.2	pres notPreg	1.0	0.0	pres notPreg	0.0	0-0	pres notPreg
DL15-188	2017-06-17	949	pres preg	0.0	1.0	pres preg	0.9	0.7-1	pres preg
DL15-193	2018-10-17	0.9	pres notPreg	1.0	0.0	pres notPreg	0.0	0-0.1	pres notPreg
DL15-197	-	715	pres preg	0.0	1.0	pres preg	0.9	0.7-1	pres preg
**DL15-244**	**-**	**112**	**pres notPreg**	**0.3**	**0.7**	**pres preg**	**0.5**	**0.3-0.8**	**pres preg**
DL15-246	-	4.8	pres notPreg	1.0	0.0	pres notPreg	0.1	0-0.2	pres notPreg
DL15-249	-	1011	pres preg	0.0	1.0	pres preg	0.9	0.7-1	pres preg
DL15-250	-	0.9	pres notPreg	1.0	0.0	pres notPreg	0.0	0-0.1	pres notPreg
DL15-252	-	620	pres preg	0.0	1.0	pres preg	0.9	0.7-1	pres preg
DL15-266	2015-07-23	0.4	pres notPreg	1.0	0.0	pres notPreg	0.0	0-0	pres notPreg
DL15-Quaqtaq1	2015-11-09	424	pres preg	0.0	1.0	pres preg	0.8	0.6-1	pres preg
DL16-013	2016-07-09	1291	pres preg	0.0	1.0	pres preg	0.9	0.8-1	pres preg
DL16-017	2016-07-09	699	pres preg	0.0	1.0	pres preg	0.9	0.7-1	pres preg
DL16-105	2016-11-11	0.2	pres notPreg	1.0	0.0	pres notPreg	0.0	0-0	pres notPreg
DL16-118	2016-06-22	777	pres preg	0.0	1.0	pres preg	0.9	0.7-1	pres preg
DL16-137	2016-06-30	647	pres preg	0.0	1.0	pres preg	0.9	0.7-1	pres preg
DL16-140	2016-07-09	0.8	pres notPreg	1.0	0.0	pres notPreg	0.0	0-0.1	pres notPreg
DL16-144	2017-06-30	1.5	pres notPreg	1.0	0.0	pres notPreg	0.0	0-0.1	pres notPreg
DL16-279	2016-07-28	0.3	pres notPreg	1.0	0.0	pres notPreg	0.0	0-0	pres notPreg
DL16-289	2016-07-05	405	pres preg	0.0	1.0	pres preg	0.8	0.6-0.9	pres preg
DL17-063	2019-06-27	264	pres preg	0.0	1.0	pres preg	0.7	0.5-0.9	pres preg
DL17-114	2017-10-08	2.8	pres notPreg	1.0	0.0	pres notPreg	0.0	0-0.1	pres notPreg
DL17-123	2017-06-26	930	pres preg	0.0	1.0	pres preg	0.9	0.7-1	pres preg
DL17-125	2017-07-03	1500	pres preg	0.0	1.0	pres preg	0.9	0.8-1	pres preg
DL17-154	2017-06-28	1.3	pres notPreg	1.0	0.0	pres notPreg	0.0	0-0.1	pres notPreg
DL17-232	2018-06-23	431	pres preg	0.0	1.0	pres preg	0.8	0.6-1	pres preg

DL17-244	2017-06-22	11.1	pres notPreg	1.0	0.0	pres notPreg	0.1	0-0.3	pres notPreg
DL17-315	2017-07-08	3.4	pres notPreg	1.0	0.0	pres notPreg	0.0	0-0.1	pres notPreg
DL17-321	2017-07-24	601	pres preg	0.0	1.0	pres preg	0.8	0.6-1	pres preg
DL17-339	2017-06-27	12.2	pres notPreg	1.0	0.0	pres notPreg	0.1	0-0.3	pres notPreg
DL17-366	2019-06-15	782	pres preg	0.0	1.0	pres preg	0.9	0.7-1	pres preg
DL18-110	2018-07-10	1217	pres preg	0.0	1.0	pres preg	0.9	0.7-1	pres preg
DL18-132	2018-07-15	1448	pres preg	0.0	1.0	pres preg	0.9	0.8-1	pres preg
DL18-148	2018-07-11	666	pres preg	0.0	1.0	pres preg	0.9	0.7-1	pres preg
DL18-179	2018-07-23	0.2	pres notPreg	1.0	0.0	pres notPreg	0.0	0-0	pres notPreg
DL18-186	2018-10-29	1271	pres preg	0.0	1.0	pres preg	0.9	0.8-1	pres preg
DL18-214	2018-10-16	0.4	pres notPreg	1.0	0.0	pres notPreg	0.0	0-0	pres notPreg
DL18-268	2018-07-07	0.2	pres notPreg	1.0	0.0	pres notPreg	0.0	0-0	pres notPreg
DL18-269	2019-08-03	0.2	pres notPreg	1.0	0.0	pres notPreg	0.0	0-0	pres notPreg
DL18-312	2018-11-14	18.0	pres notPreg	1.0	0.0	pres notPreg	0.2	0.1-0.4	pres notPreg
DL18-334	2018-11-14	0.9	pres notPreg	1.0	0.0	pres notPreg	0.0	0-0.1	pres notPreg
DL19-055	2019-07-04	0.2	pres notPreg	1.0	0.0	pres notPreg	0.0	0-0	pres notPreg
DL19-136	2019-07-05	0.9	pres notPreg	1.0	0.0	pres notPreg	0.0	0-0.1	pres notPreg
DL19-141	2019-07-02	0.9	pres notPreg	1.0	0.0	pres notPreg	0.0	0-0.1	pres notPreg
DL19-201	2019-07-05	0.7	pres notPreg	1.0	0.0	pres notPreg	0.0	0-0.1	pres notPreg
DL19-202	2019-07-05	24.8	pres notPreg	1.0	0.0	pres notPreg	0.2	0.1-0.4	pres notPreg
DL19-203	2019-07-05	958	pres preg	0.0	1.0	pres preg	0.9	0.7-1	pres preg
DL19-290	2019-11-13	0.2	pres notPreg	1.0	0.0	pres notPreg	0.0	0-0	pres notPreg
DL2011-015	2011-07-03	2.5	pres notPreg	1.0	0.0	pres notPreg	0.0	0-0.1	pres notPreg
DL2011-143	2011-11-09	1117	pres preg	0.0	1.0	pres preg	0.9	0.7-1	pres preg
DL2011-152	2011-10-10	856	pres preg	0.0	1.0	pres preg	0.9	0.7-1	pres preg
DL2011-198	2011-06-12	1.8	pres notPreg	1.0	0.0	pres notPreg	0.0	0-0.1	pres notPreg
DL2011-201	2011-07-10	0.1	pres notPreg	1.0	0.0	pres notPreg	0.0	0-0	pres notPreg
DL2011-204	2011-06-18	599	pres preg	0.0	1.0	pres preg	0.8	0.6-1	pres preg
DL2011-215	2011-06-18	1798	pres preg	0.0	1.0	pres preg	0.9	0.8-1	pres preg
DL2011-216	2011-06-09	1589	pres preg	0.0	1.0	pres preg	0.9	0.8-1	pres preg
DL2011-288	2011-06-17	0.9	pres notPreg	1.0	0.0	pres notPreg	0.0	0-0.1	pres notPreg
**DL2011-st-04**	**2011-06-23**	**112**	**pres notPreg**	**0.3**	**0.7**	**pres preg**	**0.5**	**0.3-0.8**	**pres preg**
DL2013-Sani-01	2013-06-07	421	pres preg	0.0	1.0	pres preg	0.8	0.6-1	pres preg
DLN01-10	2001-08-04	1.5	pres notPreg	1.0	0.0	pres notPreg	0.0	0-0.1	pres notPreg
DLN01-11	2001-08-04	1.3	pres notPreg	1.0	0.0	pres notPreg	0.0	0-0.1	pres notPreg

Table B 7 Estimated probability of being pregnant for St. Lawrence Estuary beluga biopsied between 2013 and 2016, Quebec, Canada, based on blubber progesterone concentration corrected for sample lipid content. Three statistical approaches were compared to assign a probability of being pregnant: a 150 ng g^{-1} threshold, a model-based clustering, and a logistic regression of progesterone concentrations (ng g^{-1} of lipid). For each animal, it is reported progesterone concentration (ng g^{-1} of lipid), the probability of a whale to be non-pregnant or pregnant, and reproductive status based on model classification for each statistical approach. All samples were from females and were of unknown reproductive status. Bold characters indicate different classifications.

**Sample**	**Sampling date**	**Colour**	**% lipid**	**Progesterone**	**150 ng g^{-1} threshold**	**Mixture model**			**Logistic regression**		
				**(ng g^{-1} lipid)**	**Classification**	**Prob. notPreg**	**Prob. pregnant**	**Classification**	**Mean Prob.**	**95% CI**	**Classification**
**DLB1312**	2013-09-04	**white**	**12.7**	**119**	**pres notPreg**	**0.9**	**0.1**	**pres notPreg**	**0.6**	**0.3-0.8**	**pres preg**
DLB1314	2013-09-06	white	27.6	589	pres preg	0.1	0.9	pres preg	0.8	0.6-1	pres preg
DLB1315	2013-09-06	offwhite	12.9	1186	pres preg	0.1	0.9	pres preg	0.9	0.7-1	pres preg
DLB1321	2013-09-10	offwhite	42.0	743	pres preg	0.1	0.9	pres preg	0.9	0.7-1	pres preg
DLB1322	2013-09-13	offwhite	4.9	2984	pres preg	0.3	0.7	pres preg	1.0	0.8-1	pres preg
DLB1326	2013-09-16	white	20.3	1085	pres preg	0.1	0.9	pres preg	0.9	0.7-1	pres preg
**DLB1327**	2013-09-16	**white**	**55.8**	**246**	**pres preg**	**0.5**	**0.5**	**pres notPreg**	**0.7**	**0.5-0.9**	**pres preg**
DLB1403	2014-09-05	white	1.8	30.8	pres notPreg	1.0	0.0	pres notPreg	0.3	0.1-0.5	pres notPreg
DLB1404	2014-09-05	offwhite	3.9	606	pres preg	0.1	0.9	pres preg	0.8	0.6-1	pres preg
DLB1406	2014-09-05	grey	49.2	434	pres preg	0.2	0.8	pres preg	0.8	0.6-1	pres preg
DLB1407	2014-09-05	white	26.1	438	pres preg	0.1	0.9	pres preg	0.8	0.6-1	pres preg
DLB1408	2014-09-05	white	5.1	18.9	pres notPreg	1.0	0.0	pres notPreg	0.2	0-0.4	pres notPreg
**DLB1409**	2014-09-05	**grey**	**14.9**	**172**	**pres preg**	**0.8**	**0.2**	**pres notPreg**	**0.6**	**0.4-0.9**	**pres preg**
DLB1410	2014-09-07	grey	18.8	3.9	pres notPreg	1.0	0.0	pres notPreg	0.1	0-0.2	pres notPreg
DLB1412	2014-09-07	white	47.1	891	pres preg	0.1	0.9	pres preg	0.9	0.7-1	pres preg
DLB1415	2014-09-08	offwhite	25.1	283	pres preg	0.4	0.6	pres preg	0.7	0.5-0.9	pres preg
DLB1416	2014-09-08	white	60.1	256	pres preg	0.5	0.5	pres preg	0.7	0.5-0.9	pres preg
DLB1417	2014-09-08	white	3.1	637	pres preg	0.1	0.9	pres preg	0.8	0.6-1	pres preg
DLB1418	2014-09-09	white	2.9	710	pres preg	0.1	0.9	pres preg	0.9	0.7-1	pres preg
DLB1419	2014-09-09	white	6.7	285	pres preg	0.4	0.6	pres preg	0.7	0.5-0.9	pres preg
DLB1422	2014-09-10	white	5.3	73.6	pres notPreg	1.0	0.0	pres notPreg	0.4	0.2-0.7	pres notPreg
DLB1423	2014-09-10	offwhite	4.9	1244	pres preg	0.1	0.9	pres preg	0.9	0.7-1	pres preg
DLB1424	2014-09-10	white	1.9	1358	pres preg	0.1	0.9	pres preg	0.9	0.7-1	pres preg
DLB1425	2014-09-10	white	13.9	3.3	pres notPreg	1.0	0.0	pres notPreg	0.0	0-0.1	pres notPreg
DLB1427	2014-09-10	grey	22.6	1077	pres preg	0.1	0.9	pres preg	0.9	0.7-1	pres preg
DLB1428	2014-09-10	offwhite	53.2	501	pres preg	0.1	0.9	pres preg	0.8	0.6-1	pres preg
DLB1429	2014-09-13	grey	34.8	3.9	pres notPreg	1.0	0.0	pres notPreg	0.1	0-0.2	pres notPreg

**DLB1430**	2014-09-13	**white**	**44.6**	**119**	**pres notPreg**	**0.9**	**0.1**	**pres notPreg**	**0.6**	**0.3-0.8**	**pres preg**
DLB1440	2014-09-15	offwhite	19.2	6.2	pres notPreg	1.0	0.0	pres notPreg	0.1	0-0.2	pres notPreg
DLB1441	2014-09-15	white	58.0	651	pres preg	0.1	0.9	pres preg	0.8	0.6-1	pres preg
DLB1443	2014-09-15	white	3.6	31.0	pres notPreg	1.0	0.0	pres notPreg	0.3	0.1-0.5	pres notPreg
DLB1452	2014-09-17	white	1.3	38.3	pres notPreg	1.0	0.0	pres notPreg	0.3	0.1-0.5	pres notPreg
DLB1520	2015-09-11	white	5.2	10.0	pres notPreg	1.0	0.0	pres notPreg	0.1	0-0.3	pres notPreg
DLB1522	2015-09-12	white	5.5	16.4	pres notPreg	1.0	0.0	pres notPreg	0.2	0-0.4	pres notPreg
DLB1524	2015-09-12	white	1.7	42.7	pres notPreg	1.0	0.0	pres notPreg	0.3	0.1-0.6	pres notPreg
DLB1526	2015-09-12	grey	2.3	1040	pres preg	0.1	0.9	pres preg	0.9	0.7-1	pres preg
DLB1527	2015-09-12	white	7.9	831	pres preg	0.1	0.9	pres preg	0.9	0.7-1	pres preg
DLB1529	2015-09-17	grey	4.1	28.6	pres notPreg	1.0	0.0	pres notPreg	0.3	0.1-0.5	pres notPreg
DLB1530	2015-09-18	offwhite	9.6	629	pres preg	0.1	0.9	pres preg	0.8	0.6-1	pres preg
DLB1555	2015-09-24	grey	51.6	0.6	pres notPreg	1.0	0.0	pres notPreg	0.0	0-0.1	pres notPreg
DLB1558	2015-09-24	white	63.7	479	pres preg	0.1	0.9	pres preg	0.8	0.6-1	pres preg
DLB1610	2016-09-09	offwhite	35.5	4.5	pres notPreg	1.0	0.0	pres notPreg	0.1	0-0.2	pres notPreg
DLB1612	2016-09-09	white	30.7	1356	pres preg	0.1	0.9	pres preg	0.9	0.7-1	pres preg
DLB1614	2016-09-10	grey	27.3	1122	pres preg	0.1	0.9	pres preg	0.9	0.7-1	pres preg
**DLB1617**	2016-09-12	**grey**	**26.4**	**177**	**pres preg**	**0.8**	**0.2**	**pres notPreg**	**0.6**	**0.4-0.9**	**pres preg**
DLB1618	2016-09-12	offwhite	39.3	1179	pres preg	0.1	0.9	pres preg	0.9	0.7-1	pres preg
DLB1620	2016-09-13	white	41.3	650	pres preg	0.1	0.9	pres preg	0.8	0.6-1	pres preg
DLB1621	2016-09-13	white	2.3	63.2	pres notPreg	1.0	0.0	pres notPreg	0.4	0.2-0.7	pres notPreg
DLB1622	2016-09-15	white	36.0	25.8	pres notPreg	1.0	0.0	pres notPreg	0.2	0.1-0.4	pres notPreg
DLB1624	2016-09-15	white	43.3	484	pres preg	0.1	0.9	pres preg	0.8	0.6-1	pres preg
DLB1628	2016-09-17	offwhite	30.7	2.8	pres notPreg	1.0	0.0	pres notPreg	0.0	0-0.1	pres notPreg
DLB1631	2016-09-19	white	31.5	7.3	pres notPreg	1.0	0.0	pres notPreg	0.1	0-0.2	pres notPreg
DLB1632	2016-09-19	grey	1.1	1283	pres preg	0.1	0.9	pres preg	0.9	0.7-1	pres preg
DLB1633	2016-09-19	grey	39.6	600	pres preg	0.1	0.9	pres preg	0.8	0.6-1	pres preg
DLB1634	2016-09-19	grey	31.7	27.3	pres notPreg	1.0	0.0	pres notPreg	0.2	0.1-0.5	pres notPreg
**DLB1635**	2016-09-20	**white**	**58.1**	**90.7**	**pres notPreg**	**1.0**	**0.0**	**pres notPreg**	**0.5**	**0.3-0.7**	**pres notPreg**
DLB1639	2016-09-21	white	18.9	978	pres preg	0.1	0.9	pres preg	0.9	0.7-1	pres preg
DLB1641	2016-09-21	grey	44.5	1.3	pres notPreg	1.0	0.0	pres notPreg	0.0	0-0.1	pres notPreg
DLB1642	2016-09-22	white	10.5	5.6	pres notPreg	1.0	0.0	pres notPreg	0.1	0-0.2	pres notPreg
DLB1646	2016-09-22	white	24.4	70.4	pres notPreg	1.0	0.0	pres notPreg	0.4	0.2-0.7	pres notPreg
DLB1647	2016-09-22	white	47.2	64.4	pres notPreg	1.0	0.0	pres notPreg	0.4	0.2-0.7	pres notPreg
DLB1648	2016-09-22	white	50.9	484	pres preg	0.1	0.9	pres preg	0.8	0.6-1	pres preg
DLB1649	2016-09-22	grey	26.7	2.2	pres notPreg	1.0	0.0	pres notPreg	0.0	0-0.1	pres notPreg
DLB1650	2016-09-22	white	6.2	1080	pres preg	0.1	0.9	pres preg	0.9	0.7-1	pres preg

**DLB1652**	**2016-09-22**	**white**	**21.4**	**185**	**pres preg**	**0.7**	**0.3**	**pres notPreg**	**0.7**	**0.4-0.9**	**pres preg**
